# A Generic Antibody-Blocking Protein That Enables pH-Switchable Activation of Antibody Activity

**DOI:** 10.1021/acschembio.3c00449

**Published:** 2023-12-18

**Authors:** Lieuwe Biewenga, Robin Vermathen, Bas J.H.M. Rosier, Maarten Merkx

**Affiliations:** † Laboratory of Chemical Biology, Department of Biomedical Engineering, Eindhoven University of Technology, 5600 MB Eindhoven, The Netherlands; ‡ Institute for Complex Molecular Systems, Eindhoven University of Technology, 5600 MB Eindhoven, The Netherlands

## Abstract

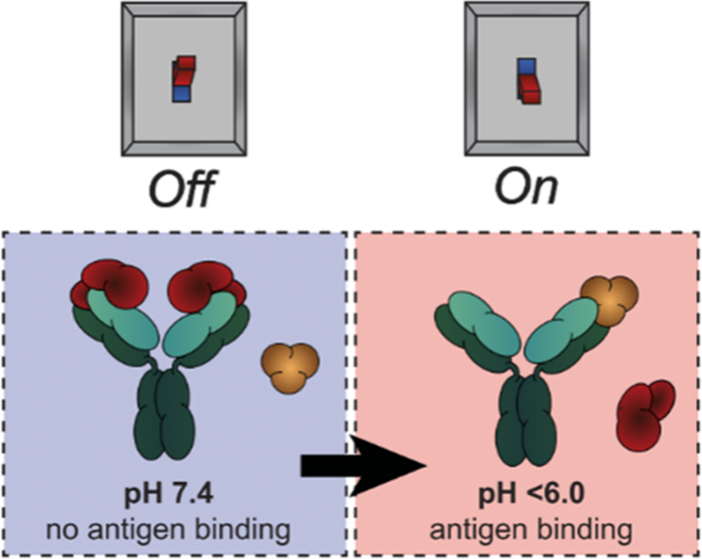

Molecular strategies that allow for reversible control of antibody activity have drawn considerable interest for both therapeutic and diagnostic applications. Protein M is a generic antibody-binding protein that binds to the Fv domain of IgGs and, in doing so, blocks antigen binding. However, the dissociation of protein M is essentially irreversible, which has precluded its use as an antibody affinity reagent and molecular mask to control antibody activity. Here, we show that introduction of 8 histidine residues on the Fv binding interface of protein M results in a variant that shows pH-switchable IgG binding. This protein M-8his variant provides an attractive and universal affinity resin for the purification of IgGs, antibody fragments (Fab and single-chain variable fragments (scFv)), and antibody conjugates. Moreover, protein M-8his enables the pH-dependent blocking of therapeutic antibodies, allowing the selective targeting of cells at pH 6.0.

## Introduction

Immunoglobulin G-type antibodies are the most common type of antibodies found in human serum.^1^ Due to their high affinity and specificity for a particular antigen, IgGs have become indispensable reagents in diagnostics and human(ized) IgGs that target disease-related antigens have become an important class of pharmaceuticals.^2,3^ Despite their excellent binding properties, antibody-based targeting can still be hampered by background binding to target antigens present in healthy cells. Therefore, in recent years, several strategies have been reported to develop activatable antibodies, antibodies whose antigen-binding sites are masked such that antibody binding is only restored in the presence of a specific molecular cue.^4−6^

A well-studied approach to engineering protease-activatable antibodies is by tethering an epitope mimetic to the antibody via a protease-cleavable linker. This strategy has been employed to construct protease-activatable antibodies for several tumor-related antigens including vascular cell-adhesion molecule 1 (VCAM-1), EGFR and PD-L1.^7−9^ An alternative design is the fusion of dimerizing leucine-zipper domains to the N-termini of the heavy and light chains,^10^ an approach that is particularly useful in case there are no suitable epitope mimetics available. pH-dependent antibody binding represents another strategy to prevent off-target toxicities and direct therapeutic antibodies specifically to the more acidic tumor microenvironment that results from increased lactate secretion by tumor cells, the so-called ‘Warburg effect’.^11,12^ Structure-based antibody engineering has been used to develop antibodies that show increased antigen affinity at low pH,^13^ but this approach requires cumbersome protein engineering for every new antibody. An alternative approach is the use of bivalent peptide-DNA locks that disassemble into weakly binding peptide monomers under acidic conditions.^14^ While these bivalent peptide-DNA locks do not require genetic or chemical modification of the antibody,^14−16^ this approach still requires the availability of peptide epitopes or mimitopes that bind the specific antibody with sufficient affinity.

Bacteria express a variety of general IgG-binding proteins that play a role in immune invasion, some of which have found widespread application in antibody purification, immobilization, and conjugation.^17,18^ Well-known examples are protein A (*Staphylococcus aureus*) and protein G (*Streptococcus sp.*) that bind to the Fc domain of IgGs from several species and protein L (*Peptostreptococcus magnus*) that targets certain subtypes of the Fv domain of the kappa light chain.^17^ More recently, a 50 kDa immunoglobulin-binding protein with remarkable binding properties was discovered in *Mycoplasma genitalium*. This protein M binds to constant regions of the Fv light chain^19^ and has a very broad scope, binding to kappa and lambda light chains of human IgG, IgA, and IgM molecules as well as murine, rat, rabbit, goat, and bovine IgGs. Unlike other IgG-binding proteins, binding of protein M sterically blocks the antigen-binding site through its C-terminal domain, which extends over the antigen-binding site.^20^

The unique dual feature of protein M, binding strongly to the variable domains of a broad range of antibodies and blocking antigen binding through an extended C-terminal domain, motivated us to explore its potential as a versatile tool for antibody purification and antibody blocking. Dissociation of the complex between IgG and wild-type protein M is very slow and essentially irreversible unless very harsh conditions are employed. We therefore set out to develop protein M variants that show pH-dependent antibody binding by systematic, structure-guided introduction of histidine residues at the IgG-binding interface of protein M. A variant containing 8 additional histidine residues (M-8his) was obtained that showed attenuated and reversible IgG binding at low pH, while retaining the high affinity and broad binding scope of the wild-type protein M at pH 7.5. This new protein M variant can be used for the purification of a wide range of antibody formats (IgGs, F(ab′)_2_, Fab, scFv) and antibody conjugates, providing clear advantages over current affinity-based purification methods. Finally, we show that protein M-8his can be used in a simple, generic, and cloning-free strategy to make pH-responsive antibodies that specifically target cell-surface receptors at pH 6.

## Results and Discussion

### Protein M Dissociation from IgG Is Very Slow

Grover et al. reported that while the presence of antigen could prevent binding of protein M, protein M could not be effectively displaced by the addition of antigen once bound to IgG, suggesting that protein M dissociates very slowly.^19^ We verified this hypothesis using surface plasmon resonance (SPR) experiments to study the kinetics of the interaction between wild-type protein M and a Fab fragment of the human IgG1 antibody cetuximab immobilized on an SPR surface. These initial experiments confirmed that protein M dissociates very slowly, showing a dissociation rate of 5.9 × 10^–5^ s^–1^ at pH 7.5 (*t*_1/2_ = 3.3 h; Table S2) and an overall *K*_D_ of 0.75 nM that is consistent with the original study by Grover et al. While this slow dissociation rate assures effective blocking of antibody activity, it also hampers reversible control of antibody activity using protein M. To find conditions that promote dissociation of protein M from IgGs, we immobilized protein M on superparamagnetic beads via sulfo-SMCC coupling using a C-terminal cysteine as a conjugation tag. As expected, these protein-M-functionalized beads effectively captured cetuximab at neutral pH, with no significant release of bound antibody upon repeated washing. Elution at pH 3.0, a standard elution condition for protein A affinity resins, also did not result in a significant release of antibody.^21,22^ Basic conditions (100 mM glycine pH 10.0) and high-salt conditions (PBS + 3 M NaCl or PBS + 3 M KCl) were equally unsuccessful. Significant dissociation of cetuximab from the superparamagnetic beads was only observed at pH < 1.5 at RT or at pH 2.2 at 55 °C (Figure S1). However, protein-M-functionalized superparamagnetic beads lost all binding capacity under these conditions, suggesting irreversible denaturation of protein M.

### Engineering of pH-Switchable Protein M Variants

**Figure 1:**
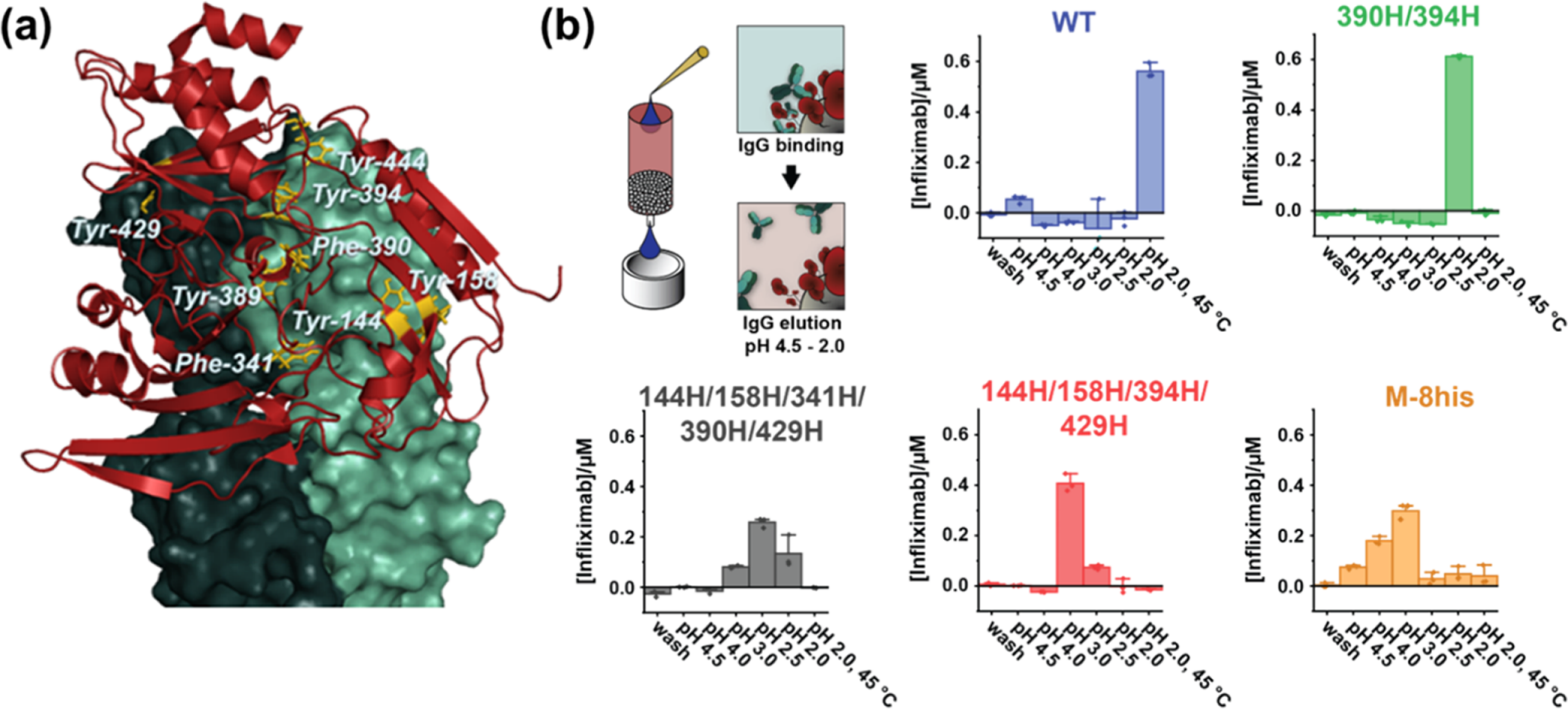
(a) Protein M (red) in complex with a Fab fragment (PDB: 4nzr[19]). The heavy chain is colored dark green, the light chain light green, and residues mutated to His yellow. (b) Elution of infliximab from protein-M-variant-functionalized superparamagnetic beads at various pHs. Protein-M-variant-functionalized superparamagnetic beads were incubated with 20 μL of 2 μM infliximab for 30 min. Subsequently, infliximab was eluted by incubating the beads in buffer with decreasing pH for 2 min. The concentration of infliximab in each elution fraction was determined by a Bradford protein assay.

The introduction of ionizable amino acids represents a general strategy to attenuate protein–protein interactions under acidic conditions.^13,23−25^ We reasoned that introduction of histidine residues on the IgG-binding interface of protein M might yield protein M variants that more readily dissociate from IgGs under mildly acidic conditions, as the imidazole side group of histidine residues has a p*K*_a_ of ∼6.0. As histidine structurally resembles phenylalanine and tyrosine, we identified 8 phenylalanine or tyrosine residues that interact with the antibody based on the reported crystal structure (Figure 1).^19^ Using multisite Quikchange mutagenesis, protein M variants were constructed containing on average 3 histidine substitutions. Three target residues are in close proximity to each other (Tyr-389, Phe-390, and Tyr-394) and could not be targeted simultaneously with this approach. Therefore, initially, three libraries were constructed targeting Tyr-389, Phe-390, or Tyr-394 in combination with the other phenylalanine or tyrosine residues.

To identify pH-switchable protein-M variants, we used a screening procedure based on the binding of protein M to cetuximab immobilized on superparamagnetic beads. The small luciferase NanoLuc was cloned N-terminally of protein M to function as a simple readout for binding and release. Four members of each library were expressed and tested for elution from cetuximab at pH 4.0, 3.0, 2.5, and 2.0. Encouragingly, all variants except two readily dissociated from cetuximab at pH 2.0 at RT and some variants even showed partial dissociation at pH 4.0 (Figure S2). Sequence analysis indicated that particularly histidine substitutions at positions 390 and 394 were associated with elution at pH > 2. Therefore, we constructed protein M F390H/Y394H to test its pH-switching behavior. In addition, since none of the mutations themselves seemed to abolish binding of protein M to cetuximab, we constructed a variant of protein M containing histidines at all 8 targeted positions (protein M Y144H/Y158H/F341H/Y389H/F390H/Y394H/Y429H/Y444H, referred to as protein M-8his). DNA encoding for these variants, together with two promising variants from the screening, was cloned in a pET28a backbone containing a C-terminal cysteine. All four constructs were expressed, purified, and coupled to superparamagnetic beads to compare their performance in IgG-affinity chromatography. Functionalized beads were incubated with an excess of infliximab, a TNFα-binding IgG. Infliximab only eluted from wild-type protein-M-functionalized beads at pH 2.0 and heating to 45 °C, similar to what was observed for cetuximab before. Beads functionalized with M-F390H/Y394H showed elution of infliximab at RT at pH 2.0, while beads functionalized with M-144H/158H/341H/390H/429H and M-144H/158H/394H/429H eluted infliximab primarily at pH 2.5–3.0 (Figure 1). The mildest elution conditions were found for protein M-8his, which showed substantial elution of infliximab at pH 4.0, on par with the conditions typically used for protein A or protein G affinity resins.^21,22^

### Affinity Purification Using Protein M-8his

**Figure 2:**
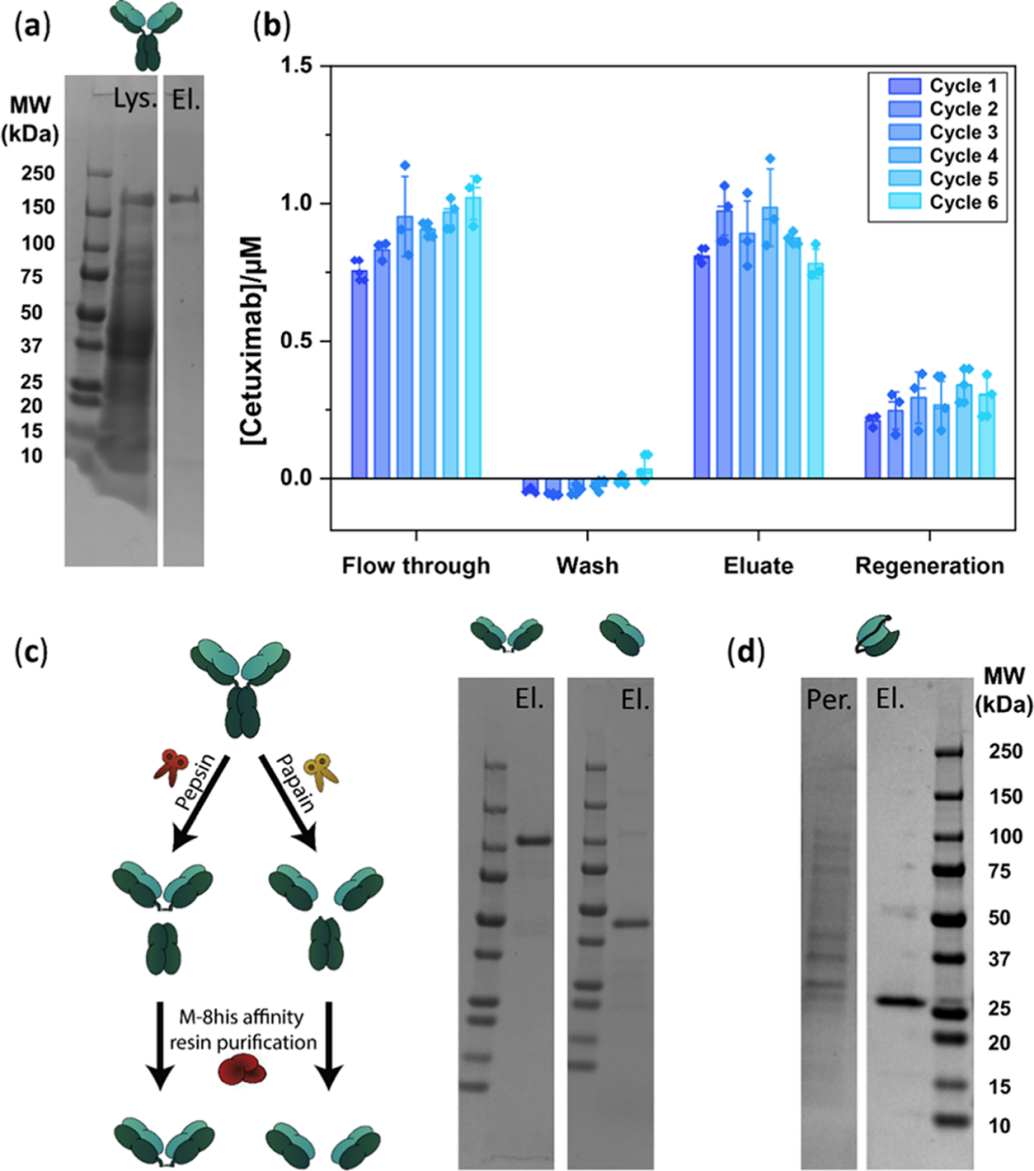
Purification of IgG, F(ab′)_2_, Fab and ScFv’s using M-8his affinity resin. (a) Nonreducing SDS PAGE gel of full-length cetuximab (150 kDa) spiked in bacterial lysate (Lys.) and M-8his affinity resin purified cetuximab (El.) (b) Reusability of the M-8his affinity resin. For 6 cycles, an excess cetuximab was incubated with M-8his affinity resin. The bound fraction was eluted with 40 mM sodium formate pH 4.0, followed by a regeneration step with 40 mM sodium formate pH 2.5. The cetuximab concentration in each fraction was determined by a Bradford protein assay. Error bars indicate standard deviation of three technical replicates. (c) Nonreducing SDS PAGE gel of F(ab′)_2_ and Fab purified with M-8his affinity resin after digestion of Cetuximab with pepsin and papain respectively. (d) Nonreducing SDS PAGE gel of periplasmic extraction from *Escherichia coli* expressing anti-HER scFv (Per.) and M-8his affinity resin purification (El.).

Since protein M binds to the Fv domain of antibodies, it could potentially be used to purify antibody derivatives such as F(ab′)_2_, Fab, and scFv’s, which is not possible with the traditional Fc-binding protein A and protein G affinity resins.^26,27^ We therefore explored the use of protein M-8his covalently coupled to agarose resin as a general affinity reagent for the purification of antibodies, antibody fragments, and antibody conjugates. To allow conjugation to the agarose resin, a cysteine was introduced as the final C-terminal amino acid following the C-terminal Strep tag (Figure S13). To test its efficacy in IgG purification, we spiked cetuximab in bacterial lysate and subsequently applied it to the protein M-8his affinity resin. Highly pure cetuximab was obtained in the elution fraction, demonstrating the specificity of M-8his affinity resin for IgGs (Figure 2a). To test the reusability of the affinity resin,^28^ we monitored its binding capacity for 6 consecutive purification cycles consisting of binding of infliximab at pH 7.4, washing at pH 7.4, an elution step at pH 4.0 and a regeneration step at pH 2.5. No significant decrease in binding capacity was observed even after 6 cycles, indicating that protein M-8His is completely stable under these conditions (Figure 2b). The affinity resin was also found to be resistant to repeated 5 min washes with up to 50 mM NaOH, conditions often used for column sanitization (Figure S3).

Next, we tested the application of M-8his affinity resin for the purification of Fab and F(ab’)_2_ fragments. To this end, cetuximab was digested with pepsin or papain and subsequently purified with the M-8his affinity resin. Both Fab and F(ab’)_2_ fragments could be purified in high purity and with a 75 and 66% yield, respectively (Figure 2c). Note that under these elution conditions, some of the fragments remain on the column, as subsequent regeneration of the column at pH 2.5 showed elution of an additional 25% of antibody (see, for example, Figure 2b). Traditional protocols often make use of resin-immobilized papain due to difficulties in separating Fab fragments from papain after digestion, but M-8his affinity resin purification is also compatible with digestion by soluble papain.^29^

To establish the performance of M-8his resin in the purification of single-chain variable fragments (scFv), we produced trastuzumab-derived anti-HER2 scFv via periplasmic expression in *E. coli*.^30^ Following periplasmic extraction, a single M-8his affinity resin purification step was sufficient to yield highly pure scFv (Figure 2d). As scFvs consist of two domains interconnected via a linker, there are two formats possible: an N-terminal light chain followed by a C-terminal heavy chain or vice versa (*V*_L_ – *V*_H_ format or *V*_H_ – *V*_L_ format, respectively). Since the different spatial positioning of the linker could affect protein M binding, we determined the affinity of wild-type protein M and protein M-8his for both kappa- and lambda-light-chain scFv expressed in a *V*_H_ – *V*_L_ or *V*_L_ – *V*_H_ format using bioluminescence resonance energy transfer (BRET) between NanoLuc-protein M and Cy3- or mNeonGreen-labeled scFv. While wild-type protein M and protein M-8his each bound to kappa- and lambda-light-chain scFv of both *V*_H_ – *V*_L_ or *V*_L_ – *V*_H_ formats with similar affinities, the affinity of protein-M-8His was found to be attenuated 5- to 100-fold (*K*_d_’s 2–50 nM) compared to that of wild-type protein M (*K*_d_’s 0.2–4 nM; Figures S4 and S5, Table S1). The *K*_D_’s for scFvs with a *V*_H_ – *V*_L_ format was generally lower than for scFvs in the *V*_L_ – *V*_H_ format, suggesting that the linker in the *V*_H_ – *V*_L_ format adopts a slightly more favorable conformation for protein M binding (Table S1).

### Purification of Antibody Conjugates

**Figure 3:**
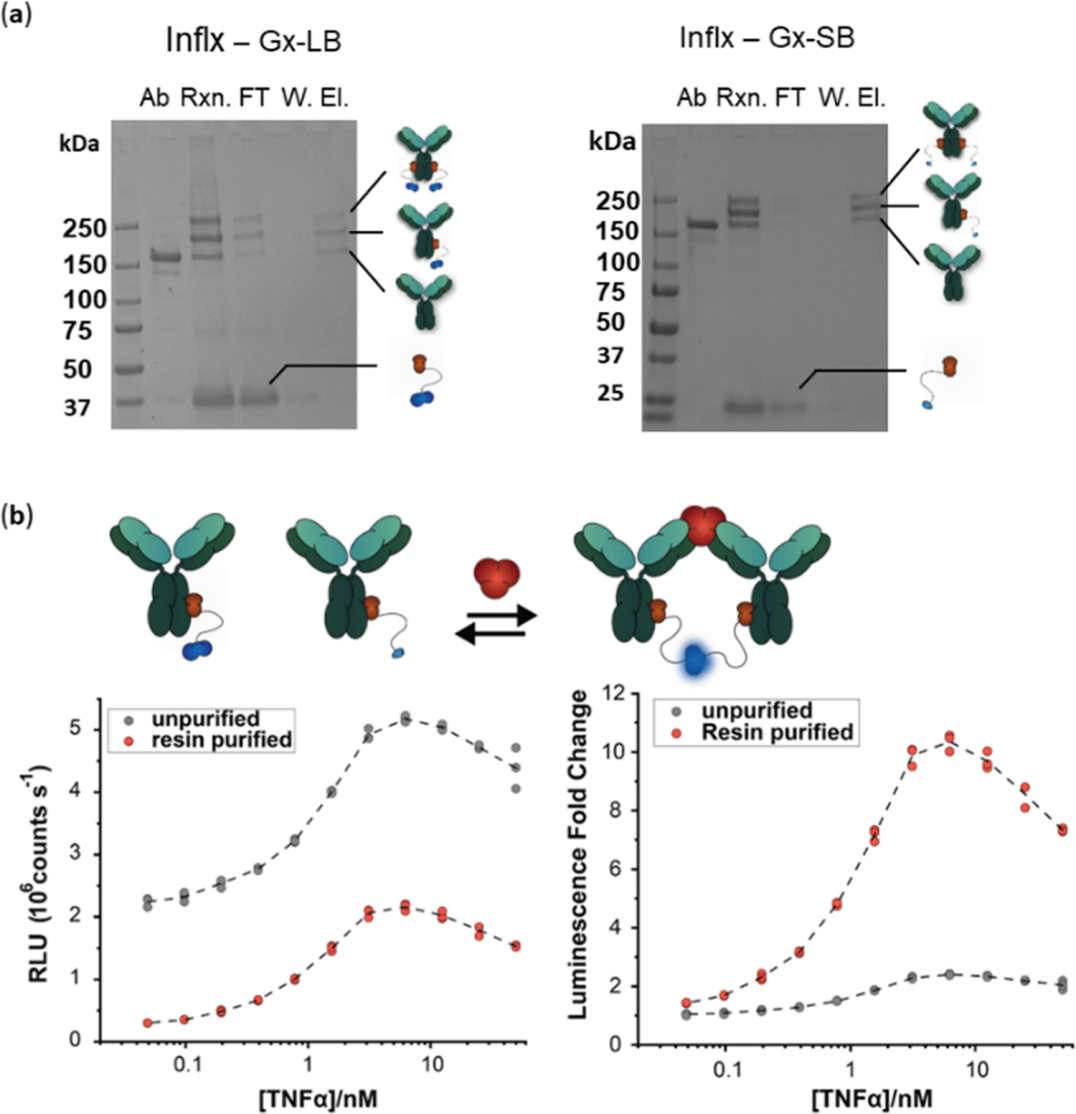
RAPPID assay with unpurified and purified infliximab-Gx-LB and Gx-SB conjugates. (a) Nonreducing SDS PAGE of the photoconjugation of 1.5 μM Infliximab with 12 μM Gx-LB and Gx-SB and subsequent purification with M-8his affinity resin. (b) Luminescence signal of purified and unpurified infliximab-Gx-LB and Gx-SB conjugates after incubation with 0–50 nM TNFα. The data are represented as the absolute luminescence signal (left) and the relative signal (right) compared to the signal in the absence of TNFα.

The functionalization of antibodies with chemical or biomolecular agents is important in applications ranging from antibody-drug conjugates to diagnostic assays. To ensure that conjugation does not interfere with antigen binding, conjugation strategies that target the Fc part of antibodies are often preferred.^31,32^ However, because some of these conjugation sites overlap with protein A and protein G binding sites, purification of antibody conjugates using traditional antibody affinity resins can be challenging.^27^ We recently developed a homogeneous sensor platform, (Ratiometric Plug-and-Play Immunodiagnostics, RAPPID) that is based on such site-specific labeling of antibodies with fusion proteins of a protein G domain (Gx), containing the photo-crosslinkable unnatural amino acid *p*-benzoyl-l-phenylalanine, and split luciferase components Large BiT (LB) and Small BiT (SB).^33^ Complete antibody conjugation typically requires an excess of Gx-LB and Gx-SB, which can contribute to the background signal and thus limit the sensor’s dynamic range. We tested whether purification of the sensor components from excess Gx-LB and Gx-SB using the protein M-8his affinity resin would improve the dynamic range of a TNFα-sensing RAPPID assay consisting of infliximab photoconjugated to either Gx-LB or Gx-SB. As TNFα is trimeric, infliximab conjugated to Gx-LB and infliximab conjugated to Gx-SB can bind to the same TNFα trimer, facilitating complexation of LB and SB which results in a luminescent signal. The photoconjugation reaction yielded a mixture of nonconjugated, single-conjugated, double-conjugated antibody and a portion of unreacted Gx-LB and Gx-SB, in line with previous observations.^33^ Part of the crude photoconjugation reaction mixture was further purified using M-8his affinity resin, which removed the unreacted Gx-LB and Gx-SB (Figure 3a). Although both purified and unpurified sensor components were capable of detecting TNFα, the unpurified sensor showed a significantly higher background luminescent signal (Figure 3b). As a result, the dynamic range of the TNFα RAPPID assay increased from 2.4-fold to 10.3-fold upon protein M-8his-mediated purification.

### Dissociation Kinetics of Protein M Variants

**Figure 4:**
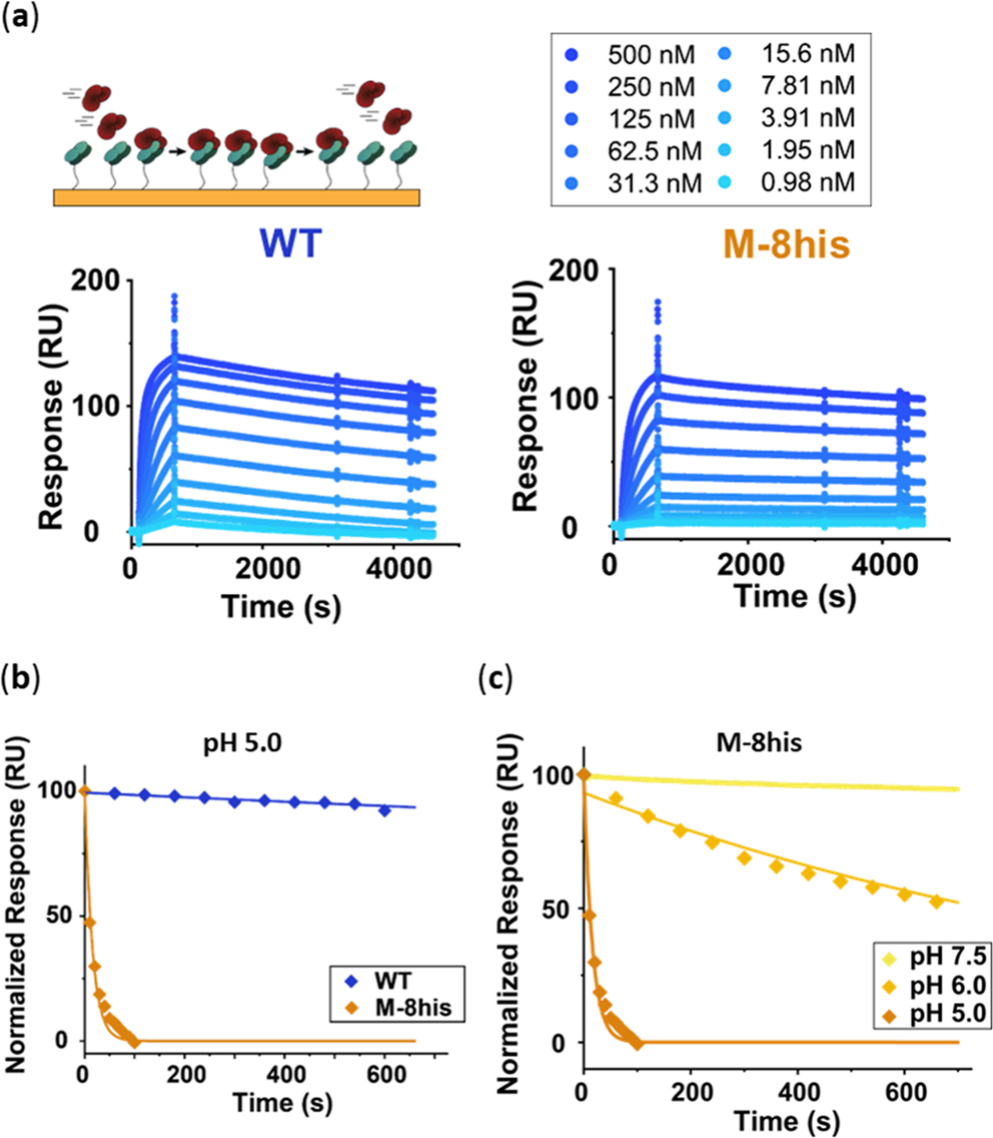
SPR analysis of the kinetics of the interaction between cetuximab Fab fragments and protein M. (a) SPR traces of binding and dissociation of protein M WT and M-8his to immobilized cetuximab Fab at pH 7.5. (b) Dissociation of protein M WT and M-8his from immobilized cetuximab Fab at pH 5.0. 500 nM protein M WT or M-8his was flown over the cetuximab Fab chip for 9 min, and the SPR signal was normalized to 100 RU for comparison. Pulses of 10 or 60 s with buffer of the indicated pH were alternated with running buffer (pH 7.5) washes to remove nonspecifically bound protein M. Individual data points and fit to eq S1 are shown. (c) Dissociation of M-8his from immobilized cetuximab Fab at pH 7.5, 6.0, and 5.0.

To provide further insight into the effect of pH on the binding properties of the pH-switchable protein M variants, surface plasmon resonance (SPR) was used to monitor association and dissociation kinetics as a function of pH. Cetuximab-derived Fab fragments were immobilized on the surface of the SPR chip, and varying concentrations of protein M variants were flown over the chip. The introduction of up to 8 mutations on the binding interface of protein M had remarkably little effect on the affinity for cetuximab at pH 7.5 with apparent *K*_D_ values ranging from 0.1 to 1 nM (Figures 4a and S6, Table S2). The *k*_off_ values of WT protein M and protein M variants were low at this pH. In fact, dissociation of several of the pH-switchable protein M variants was too slow to be accurately measured by SPR, which makes it difficult to accurately determine affinities. Direct determination of dissociation kinetics at lower pH turned out to be challenging as protein M becomes positively charged at acidic pH (protein M has a theoretical pI of 6.0) and was found to nonspecifically interact with the negatively charged SPR chip surface. We therefore adapted our SPR procedure by pulsing for short periods with low-pH buffer (pH 5.0 to 3.0) followed by a short pulse at pH 7.5 to remove nonspecifically bound protein M from the SPR chip (Figures S7–S11). For WT protein M a very modest increase in dissociation rate was observed between pH 7.5 (5.9 × 10^–5^ s^–1^) and pH 3.0 (1.9 × 10^–4^ s^–1^), which is in line with our observations that protein M only dissociates from IgGs under very harsh conditions. In contrast, protein M-8his rapidly dissociated from the chip at pH 5.0, with a dissociation rate of 6.3 × 10^–2^ s^–1^, representing a 1900-fold increase in dissociation rate compared to pH 7.5 (Figure 4c and Table S3). In fact, dissociation of protein M-8his was also found to be substantially enhanced at pH 6, showing a dissociation rate of 8.3 × 10^–4^ s^–1^ (Figure 4c and Table S3). Enhanced dissociation rates were also observed for the other protein M variants but at pH values lower than 5 (Table S3).

### pH-Triggered Activation of Therapeutic Antibodies

**Figure 5:**
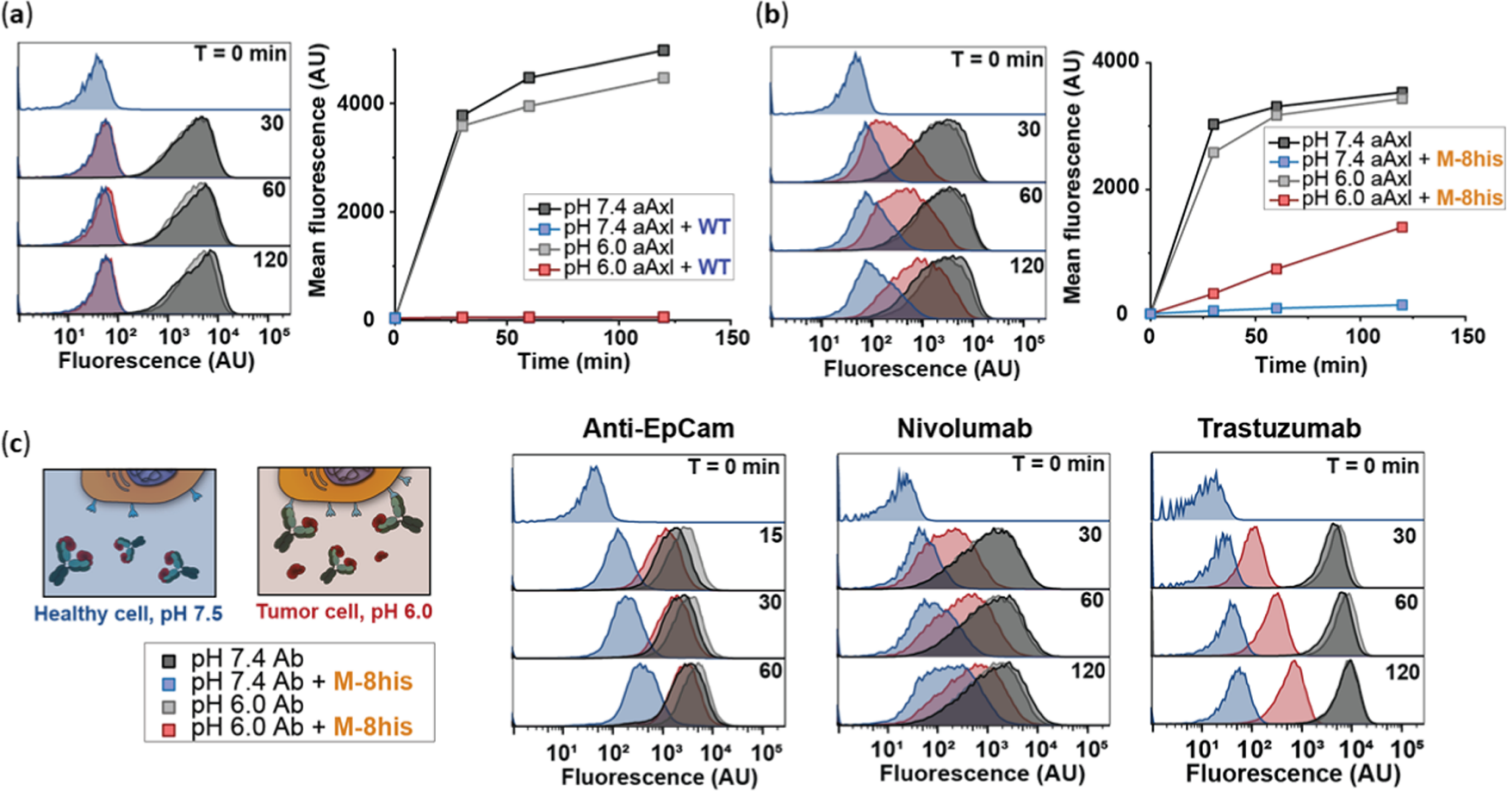
pH-induced antibody activation. FACS analysis of A431 cells labeled with (a) 1 nM aAxl and 1 nM wt protein M-bound aAxl or (b) 1 nM M-8his-bound aAxl in the presence of 100 nM IgGs at pH 7.4 or pH 6.0. (c) FACS analysis of A431, PD-1-transfected CHO, or SK-BR-3 cells labeled with anti-EpCam (5 nM) and M-8his-complexed anti-EpCam, Nivolumab (1 nM) and M-8his-complexed Nivolumab, Trastuzumab (1 nM) and M-8his-complexed Trastuzumab, respectively, at pH 7.4 or pH 6.0. All antibodies were labeled with Alexa647-NHS.

The overproduction of lactate by tumor cells has been reported to decrease the pH in the tumor microenvironment, with reported pH values ranging between pH 5.8 and 7.0 depending on among others tumor size, tumor density, and tumor type.^11,12^ We envisioned that protein M-8his might be used as a pH-activatable mask that blocks antibody activity at neutral pH (healthy tissue) and selectively activates antibodies under the acidic conditions of the tumor microenvironment. To test whether protein M-8his allows for pH-dependent receptor targeting, we used an Alexa647-labeled antibody targeting Axl, a receptor tyrosine kinase that is associated with various cancers and overexpressed on A431 tumor cells.^34,35^ The anti-Axl antibody was first incubated for 1h with a small excess of protein M-8his. Next, the blocked antibody was added to A431 cells at pH 7.4 or pH 6.0, and flow cytometry was used to monitor antibody activation and subsequent receptor binding as a function of time. As a control, we also monitored the binding of the same concentration of anti-Axl in the absence of protein M-8his. As expected, anti-Axl rapidly bound to A431 cells at pH 7.4 and pH 6.0 in the absence of protein M-8his (Figure 5b), while binding was completely blocked at pH 7.4 when using anti-Axl preincubated with protein M-8his. However, at pH 6.0, a steady increase in receptor binding was observed for the protein M-8his-complexed anti-Axl, whereas no significant activation was observed for anti-Axl complexed with WT protein M (Figure 5a). Cell binding experiments with free anti-Axl and protein-M-complexed anti-Axl were performed in the presence of a large excess (100 nM) of serum antibodies. Nonetheless, no significant anti-Axl activation was observed at pH 7.4, which indicates that antibody activation is controlled by kinetics rather than thermodynamics.

To establish the general applicability of this approach, we tested the performance of protein M-8his using a panel of antibodies including mouse (anti-EpCam), chimeric (cetuximab), humanized (trastuzumab), and fully human (nivolumab) antibodies targeting a diverse set of therapeutically relevant antigens. Unexpectedly, our strategy did not work for cetuximab. Protein M binding did not fully block the antigen-binding site in cetuximab, as evidenced by the observed colocalization of protein M and cetuximab on the cell surface of EGFR-overexpressing A431 cells (Figure S12). However, pH-responsive antibody activation was observed for all other antibodies without any further optimization (Figure 5c). There was some variation in the rate of activation at pH 6.0 between trastuzumab, nivolumab, and anti-EpCam. The highest activation rate was observed for anti-EpCam, showing almost full activation in 30 min. However, some background activation was also observed at pH 7.4 for this antibody. In contrast, the activation rates of trastuzumab and nivolumab were considerably smaller, but less background activation at pH 7.4 was also observed. These results show that protein M-8his can be used as a generic pH-switchable antibody-blocking protein but that the absolute rate of protein M dissociation is also affected by sequence variation between the different *V*_L_ domains present in trastuzumab, nivolumab, and anti-EpCam.

## Conclusions

Protein M combines the intriguing features of being a truly generic antibody-binding protein while also effectively blocking the antigen-binding site that is unique to each antibody. Another remarkable property of protein M binding is that its dissociation is very slow (*t*_1/2_ = 3.3 h), which has hampered its application as a generic reagent for antibody purification and its use as a generic building block to reversibly control antibody activity. In this work, we engineered a pH-switchable variant of protein M that unlocks both of these applications by enabling rapid dissociation at mildly acidic conditions. Protein M was found to be remarkably resilient to mutations at the binding interface, allowing substitution of up to 8 aromatic amino acids by histidines, while retaining a low nM affinity toward antibodies, antibody fragments, and antibody conjugates at neutral pH. This suggests that protein M has evolved a binding interface that can accommodate and compensate for the differences between antibody isotypes. This plasticity may also explain that introduction of multiple histidine residues was required to arrive at a protein M domain that readily dissociates at pH 4.0, although it remains to be established whether all 8 histidines contribute to the pH-dependent binding properties. The protein M-8his variant remained remarkably stable, allowing repeated cycles of binding, elution, and regeneration at pH 2.5 without any noticeable decrease in binding capacity.

Immobilized protein M-8his provides an attractive affinity resin for the purification of a broad spectrum of antibodies. In addition to binding many different antibody types and species, protein M-8his binds antibody derivatives lacking the Fc region, which provides a clear advantage over commonly used affinity resins based on protein A and protein G.^26,27^ Other proteins that bind to the Fv part of IgGs are known, but they have a more limited binding scope. For instance, protein A also binds to the *V*_H_3 domain, but this domain is present in only 30–50% of circulating IgG.^36,37^ An engineered protein G variant, protein G-A1, binds to the C_H_1 domain of the IgG1 isotype with a high affinity (*K*_D_ of 25 nM), but the affinity for the IgG2, IgG3, and IgG4 subtype was 20- to 50-fold lower.^38^ A well-known protein that is used to purify (Fab′)_2_, Fab, and scFv’s is protein L, which binds to the variable domain of antibodies. However, protein L only binds to antibodies with *V*κI, *V*κIII, and *V*κIV and also with a relatively low affinity of 100 nM.^39,40^ In contrast, protein M-8his binds to scFv’s with either a kappa or a lambda light chain with affinities of 2.1 and 15 nM, respectively.

While effective elution of antibodies from protein M-8his resin requires incubation at pH 4.0, detailed kinetics experiments revealed that the dissociation rate is already increased 20-fold at pH 6. This property was used to allow specific activation of therapeutic antibodies at pH 6 without the need for genetic manipulation and with minimal individual optimization. An interesting feature of this approach is that the pH-triggered activation is kinetically controlled, ensuring efficient blockage of therapeutic antibodies at neutral pH for several hours, even in the presence of a large excess of competing IgGs. While protein M is able to block antigen binding in the vast majority of antibodies tested by us and others,^19^ cetuximab was found to be an interesting exception. More detailed biophysical and structural studies are required to understand how cetuximab-EGFR or cetuximab-protein M binding is different from that of other antibodies. In vitro models of the tumor microenvironment could be used to further validate the concept of using protein M-8his to confer pH sensitivity to antibody-based imaging and therapeutics. Ultimately, in vivo experiments are required to determine whether the use of pH-activatable antibodies also translates into specific accumulation and activity of antibodies in the tumor microenvironment.

## Methods

### Cloning

The pET28a(+) vector containing DNA encoding for protein M, containing an N-terminal his-tag and on the C-terminus a strep-tag followed by a cysteine, was ordered from GenScript. The sequences for scFv’s, containing a PelB leader sequence and a C-terminal His-tag were ordered as gBlocks from Integrated DNA Technologies. 500 ng of gBlock DNA was digested with NdeI and XhoI according to the manufacturer’s protocol (New England Biolabs), and subsequently spin column purified (QIAquick PCR purification kit, QIAGEN). 2 μg of a pET24a was digested with NdeI and XhoI and subsequently purified from agarose gel (QIAquick gel extraction kit, QIAGEN). The gBlocks were ligated with the pET24a backbone using T4 ligase (New England Biolabs) and transformed in chemically competent *E. coli* NovoBlue cells. All cloning and mutagenesis results were confirmed by Sanger sequencing (BaseClear). The DNA and amino acid sequences can be found in Figures S13–S18.

### Library Construction and Screening

Three focused protein M libraries were designed so that each member contains a subset of histidine mutations at 6 different positions. In all libraries, positions Tyr-144, Tyr-158, Phe-341, Tyr-429, and Tyr-444 were targeted. In library 1 also Tyr-389 was targeted, in library 2, Phe-390, and in library 3, Tyr-394. The libraries were constructed using the QuikChange Lightning Multi Site-Directed Mutagenesis Kit (Agilent Technologies), using the manufacturer’s protocol. As a starting template, a pET28a vector containing NanoLuc-protein M fusion construct was used. Eight different primers were designed that contained the Y144H, Y158H, F341H, Y389H, F390H, Y394H, Y429H, or Y444H mutation. Three mixtures of 6 primers were created by mixing primers containing the Y144H, Y158H, F341H, Y429H, and Y444H mutation with either the primer containing Y389H, F390H, or Y394H mutation. 300 ng of these 3 primer mixtures (50 ng of each primer) was combined with 250 ng template vector to run 3 parallel Quikchange PCR reactions. After DpnI digestion, the reaction mixture was purified (QIAquick pcr purification kit, QIAGEN) and used to electroporate electrocompetent NovoBlue cells. Of several individual colonies, plasmid DNA was extracted and sequenced to assess the library quality. All remaining colonies were pooled, and plasmid DNA was extracted. The library plasmid DNA was used to transform *E. coli* Bl21 (DE3) cells. Individual colonies were picked and grown in 5 mL of LB medium supplemented with 50 μg/mL kanamycin at 37 °C until the culture reached an OD_600_ of 0.4. 250 μM isopropyl β-d-1-thiogalactopyramoside (IPTG) was added to the culture, and the protein was expressed overnight at 20 °C. 1 mL of the culture was harvested by centrifugation and the pellet was resuspended in 200 μL of lysis buffer (100 mM Tris pH 8.0, 150 mM NaCl, 1 mM EDTA, 1 mg mL^–1^ lysozyme, Complete protease inhibitor [Roche]). After 30 min of lysis at RT, the lysate was cleared by centrifugation and 5 μL of the lysate was mixed with 5 μL of antibody-functionalized superparamagnetic beads and 490 μL of binding buffer (100 mM Tris pH 8.0, 150 mM NaCl, 1 mM EDTA, 0.01% Tween-20, 0.5 mM TCEP) and incubated at RT for 30 min. To four different LoBind tubes (Eppendorf), 100 μL of the suspension was added and the tube was placed on a magnetic stand for 1 min to clear the suspension. The supernatant was removed and the beads were washed in 100 μL of binding buffer. The tubes were placed on a magnetic stand for 1 min and the supernatant was removed. The beads were resuspended in 40 mM sodium formate pH 4.0, 3.0, 2.5, or 2.0 and incubated at RT for 1 min. 4 μL of the suspension was mixed with 16 μL neutralization buffer (1 M Tris pH 8.5, 0.01% Tween-20) and transferred to a white 384-wells plate. The tubes were placed on the magnetic stand for 1 min and 4 μL of the supernatant was mixed with 16 μL of neutralization buffer and transferred to a white 384-well plate. 1 μL of 1:50 nanoglo (Promega) in neutralization buffer was added to the collected samples and the luminescence signal was recorded on a Tecan Spark 10 M plate reader.

### Protein M Purification

A colony of *E. coli* Bl21(DE3) cells freshly transformed with pET28a containing the protein M insert was used to inoculate 5 mL of LB medium supplemented with 50 μg/mL kanamycin and grown overnight at 37 °C. The overnight culture was used to inoculate 1 L of 2YT medium (5.0 g/L NaCl, 10 g/L yeast extract, 16 g/L peptone) and incubated at 37 °C until the OD_600_ reached ∼0.5. Expression was induced by the addition of IPTG to a final concentration of 250 μM. After overnight expression at 20 °C the cells were harvested by centrifugation and subsequently lysed by Bugbuster protein extraction reagent (Novagen) supplemented with benzonase for 1 h at RT. The lysate was cleared by centrifugation and subsequently filtered through a 0.2 μm filter and applied to a column containing 2 mL of His-bind resin. The column was washed with 10 column volumes (CV) of buffer A (PBS + 370 mM NaCl + 10% v/v glycerol + 20 mM imidazole, pH 7.4) and subsequently eluted with elution buffer (buffer A + 230 mM imidazole). The eluate was loaded on a 1 mL Strep-Tactin XT column. The column was washed with 5 CV strep-wash buffer (100 mM Tris pH 8.0, 150 mM NaCl, 1 mM EDTA) and eluted with strep-elution buffer (strep-wash buffer + 50 mM biotin). The purity of the eluted protein was checked by SDS PAGE. The concentration was determined by measuring the absorption at 280 nm (ε 58 220 M^–1^ cm^–1^). The protein was aliquoted, flash-frozen in liquid nitrogen, and stored at −80 °C until further use. Protein M-NanoLuc fusion protein was expressed and purified by using a similar protocol.

### Superparamagnetic Bead Functionalization

1 mL of 10 μM dimeric protein Gx with a C-terminal cysteine or protein M variant in PBS was incubated with 5 mM TCEP for 1 h at RT. The protein was desalted on a PD-10 column equilibrated in 100 mM sodium phosphate pH 7.0. 4x 150 μL of 1 μM cetuximab, 1 μM protein Gx in 100 mM sodium phosphate pH 7.0 was prepared and protein Gx was photo-cross-linked to cetuximab using a reported procedure.^1^ 0.6 mg of superparamagnetic beads (Dynabeads M-270 amine, Invitrogen) were equilibrated in PBS pH 7.2 and labeled with 1.8 μg sulfo-SMCC in PBS pH 7.2, 50% v/v DMSO, 20 μL for 30 min at RT under slow tilt and rotation. The beads were washed twice with 200 μL of 100 mM sodium phosphate at pH 7.0 and 25 μM TCEP. The beads were resuspended with the photo-cross-linked cetuximab or protein M variant and incubated for 3 h at RT with slow tilt and rotation. The coupling efficiency was estimated by measuring the absorbance at 280 nm before and after coupling. After the coupling, the beads were washed 2× with 400 μL of storage buffer (PBS + 0.01% Tween-20) and stored in 100 μL of storage buffer at 4 °C.

### Infliximab Elution from Protein M-Functionalized Superparamagnetic Beads

In triplicate, 20 μL of 2 μM infliximab (40 pmol) in PBS was incubated with protein-M-functionalized superparamagnetic beads (∼20 pmol of protein M) for 30 min at RT with slow tilt and rotation in Lobind tubes (Eppendorf). After 30 min, the tube was placed on a magnetic stand for 1 min and the supernatant was collected. The beads were resuspended with 200 μL of PBS for 2 min and subsequently placed on a magnetic stand for 1 min. 20 μL of the supernatant was collected in a 96-well plate, and the rest was discarded. The beads were resuspended with 20 μL of 40 mM sodium formate pH 4.5 for 2 min and subsequently placed on a magnetic stand for 1 min. The supernatant was collected, and the pH was neutralized by the addition of 2 μL of 1 M Tris pH 8.5. Subsequently, the beads were, respectively, resuspended with 20 μL of 40 mM sodium formate at pH 4.0, 3.0, 2.5, and 2.0 using a similar procedure. Finally, the beads were resuspended in 20 μL of 40 mM sodium formate pH 2.0 and the tube was placed in a heat block at 45 °C for 5 min and subsequently placed on a magnetic stand for 1 min. To all collected fractions, 180 μL of Bradford reagent was added and incubated for ∼5 min. A dilution series of known infliximab concentrations was included in each assay and used as a reference. The absorbance at 595 nm was measured, and based on an infliximab dilution series, the concentration of antibody in each sample was determined.

### M-8his Functionalization of Sulfolink Resin

For a typical coupling reaction, 10 mg of M-8his was thawed and TCEP was added to a final concentration of 2 mM and incubated for 45 min at RT. After 45 min incubation, 0.5 mL of sulfolink coupling resin slurry (Thermo Scientific) equilibrated in coupling buffer (50 mM Tris pH 8.5, 5 mM EDTA) was added, and the suspension was incubated for 1 h at RT under slow tilt and rotation. The suspension was transferred to a 5 mL column, and the unreacted protein M was collected. The resin was washed with 5 mL of coupling buffer. Subsequently, the resin was incubated with 3 mL of 50 mM cysteine in coupling buffer and incubated for 45 min at RT. The column was washed with 3 mL of 1 M NaCl in Milli-Q and subsequently equilibrated in storage buffer (20 mM Tris pH 8.0, 100 mM NaCl, 5 mM EDTA). The resin was removed from the column and stored in a 12 mL falcon tube as a 25% slurry in a storage buffer.

### Repeated M-8his Affinity Resin Purification

63.5 μL of 25% M-8his affinity resin slurry (corresponding to ∼1 nmol of M-8his) was added to a Pierce MicroSpin column (Thermo Fisher Scientific) and placed in a 2 mL Eppendorf tube. Excess liquid was removed by centrifugation (1500 rpm for 15 s). The affinity resin was resuspended with 100 μL of 2.5 μM cetuximab in wash buffer and incubated for 5 min while occasionally resuspending the affinity resin by pipetting. The MicroSpin column was centrifuged, and the flow-through was collected. The affinity resin was resuspended in 350 μL of wash buffer, the MicroSpin column was centrifuged, and the flow-through was collected. The affinity resin was resuspended in 50 μL of elution buffer (40 mM sodium formate pH 4.0) and incubated for 5 min. The MicroSpin column was centrifuged, and the flow-through was collected. The affinity resin was again resuspended in 50 μL of elution buffer and incubated for 5 min. The MicroSpin column was centrifuged, and the flow-through was combined with the previous fraction. The affinity resin was resuspended in 100 μL of regeneration buffer (40 mM sodium formate, pH 2.5). The MicroSpin column was centrifuged, and the flow-through was collected. The affinity resin was resuspended in 200 μL of wash buffer to neutralize the pH. The experiment was conducted in triplicate and repeated 6 times. 10 μL of all collected fractions was added to a transparent 96-well plate. 10 μL samples of a dilution series of cetuximab in wash buffer and elution buffer were also added to the 96-well plate. 190 μL of Bradford reagent was added to each well and incubated for 5 min at RT. The absorbance at 595 nm was recorded. The concentration of cetuximab in the flow-through and wash fraction was calculated from the measured absorbance of known concentrations of cetuximab in wash buffer. The concentration of cetuximab in the elution and regeneration fractions was calculated from the measured absorbance of the cetuximab dilution series in elution buffer.

### Sodium Hydroxide Resistance of M-8his Affinity Resin

To determine the resistance of M-8his affinity resin to sodium hydroxide, the MicroSpin column purification procedure (see repeated M-8his affinity resin purification) was followed with a few modifications. A single 100 μL elution step using 40 mM sodium formate, pH 3.0, as elution buffer was used. After the elution step the pH of the affinity resin was neutralized by the addition of 200 μL of wash buffer. After centrifugation, the resin was resuspended in 200 μL of sodium hydroxide (10, 50, 200, or 500 mM) and incubated for 5 min at RT. After centrifugation, the pH of the affinity resin was neutralized by the addition of 200 μL of wash buffer.

### SPR Analysis

Measurements were performed on a Biacore X100 (GE Healthcare) using HBS-EP as running buffer (10 mM HEPES pH 7.5, 150 mM NaCl, 3 mM EDTA, and 0.005% P20 [cytiva]). Channel 2 of a CM5 chip (cytiva) was functionalized with cetuximab fab fragments using EDC/NHS coupling; 75 mg/mL EDC in Milli-Q and 11.5 mg/mL NHS in Milli-Q were freshly prepared. The chip surface was activated using EDC and NHS (7 min contact time, flow of 10 μL/min). 50 nM Cetuximab Fab fragment in 10 mM sodium phosphate pH 6.0 was flown over the chip, and ∼250 RU of cetuximab Fab fragment was immobilized. The surface was quenched by flowing 1 M ethanolamine in Milli-Q (pH 8.5) over the chip for 7 min, 10 μL/min. Prior to the SPR run, protein M variants were incubated with 2 mM TCEP for 1 h at RT. After reduction, the protein was loaded on a PD-10 column equilibrated in HBS-EP. Protein M variants at concentrations ranging from 500 to 0.98 nM were flown over both channels using a flow of 10 μL/min and a 9 min contact time. The chip was washed for 1 h using running buffer (HBS-EP), followed by a regeneration step using a 60 s pulse of 10 mM glycine pH 1.5 at a flow rate of 10 μL/min. The signal from channel 1 was subtracted from the signal from channel 2. A cycle where only buffer was injected was subtracted from all runs to correct for small signal drift.

For measuring dissociation rates at pH 6.0, 5.0, 4.0, and 3.0, protein M variants at concentrations ranging from 500 to 62.5 nM were flown over both channels using a flow of 10 μL/min and a 9 min contact time. Because protein M binds nonspecifically to the CM5 chip at a low pH, an SPR procedure that involves short pulses with low-pH buffer (to dissociate protein M from the fab fragment) alternated with HBS-EP (to remove nonspecifically bound protein M) was developed. After protein M binding to immobilized Fab, 11 pulses of low-pH buffer (10 mM sodium formate, 150 mM NaCl, 3 mM EDTA, 0.005% P20 at pH 5.0, 4.0, 3.0, or 10 mM sodium phosphate, 150 mM NaCl, 3 mM EDTA, 0.005% P20 at pH 6.0) was flown over the chip with a contact time of 1 min or 10 s and a flow rate of 10 μL/min. In between each injection, HBS-EP was flown over the chip for 1 min. Finally, a regeneration 60 s pulse of 10 mM glycine pH 1.5 was flown over the chip. The signal from channel 1 was subtracted from the signal from channel 2. The 10 s average signal after the final regeneration step was subtracted from all data points. The average signal from 40 to 50 s after each pulse with low-pH buffer was recorded and fitted with eq S1, using a single *k*_off_ value.
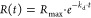
1

### Mammalian Cell Culturing

Human A431 carcinoma cells and high PD-1 expressing CHO K1^2^ cells were cultured in RPMI-1640 medium supplemented with 10% Fetal Bovine Serum (FBS, Gibco 26140) and 1% penicillin/streptomycin (Gibco 15140) in T75 flasks at 37 °C, 5% CO_2_. SK-BR-3 cells were cultured in the same medium supplemented with 1 mM sodium pyruvate. Cells were passed at 80% confluency. The cells were detached using 2 mL of 0.05% trypsin-EDTA solution (Gibco, 25300) for 5 min at 37 °C.

### pH-Selective Labeling of Cells

Anti-Axl antibody D,^3^ Cetuximab, Nivolumab, Trastuzumab, and anti-EpCam antibody (Clone 1B7, eBioscience) were labeled with AlexaFluor647-NHS ester (Invitrogen) according to the manufacturer’s instructions. A solution of 20 nM (60 nM for anti-EpCam) labeled antibody and 100 nM (300 nM for anti-EpCam) protein M (WT or M-8his) in PBS + 1 mg/mL BSA was prepared and incubated at RT for 1 h to allow for protein M complexation. Also a solution without protein M was prepared (uncomplexed). Freshly harvested mammalian cells were centrifuged, the supernatant was discarded, and the cell pellet was resuspended in PBS pH 7.4. The cells were split into 2 equal parts and centrifuged. One part was resuspended in PBS + 1 mg/mL BSA pH 7.4, and the other part was resuspended in PBS + 1 mg/mL BSA pH 6.0. To 1 mL samples of cells was added 50 μL of complexed or uncomplexed antibody and the cells were incubated in a heat block at 37 °C under 200 rpm mixing. After 30, 60, and 180 min (for anti-Epcam 15, 30, and 60 min), samples were collected and centrifuged, and the pellet was resuspended in PBS + 1 mg/mL BSA pH 7.4 and analyzed by flow cytometry. All flow cytometry measurements were performed using a BD FACS Aria III equipped with a 70 μm nozzle. Doublet cells were excluded by standard doublet discrimination with forward scatter area versus height plots. AlexaFluor647 was excited by a 633 nm laser and detected through a 660/30 bandpass filter. The fluorescence of 30,000 cells was recorded. Data were displayed using FlowJo software.

## Supporting Information Available

General reagents, generation and purification of Fab fragments, purification of IgG and scFv from cell lysates, expression and purification of adalimumab scFvs’s and CR6261-mNeonGreen ScFv’s, BRET titration experiments with scFv’s, TNFα-RAPPID assay, DNA, and protein sequences (PDF)
