# Supplementary material for: A Generic Antibody-Blocking Protein That Enables pH-Switchable Activation of Antibody Activity
Source: ACS Chem Biol. 2023 Dec 18;19(1):48–57. doi: 10.1021/acschembio.3c00449 (PMC10804362; doi:10.1021/acschembio.3c00449)
Supplement: Supplementary file 1 — cb3c00449_si_001.pdf [file cb3c00449_si_001.pdf]

Supporting information

A generic antibody-blocking protein that enables pH-switchable activation of antibody binding

Lieuwe Biewenga[1,2], Robin T. Vermathen[1,2], Bas J.H.M. Rosier[1,2], Maarten Merkx[1,2*]

[1.] Laboratory of Chemical Biology, Department of Biomedical Engineering, Eindhoven University of Technology, 5600 MB Eindhoven, The Netherlands.

[2.] Institute for Complex Molecular Systems, Eindhoven University of Technology, 5600 MB Eindhoven, The Netherlands.

*Correspondence should be addressed to m.merkx@tue.nl.

Experimental section

General reagents

Therapeutic antibodies cetuximab (Erbitux, Merck), trastuzumab (Herceptin, Roche) and nivolumab (Opdivo, Bristol-Meyers Squibb) were obtained via the Catherina Hospital pharmacy in Eindhoven, The Netherlands. Infliximab was obtained via the Máxima Medisch Centrum pharmacy in Veldhoven, The Netherlands. The anti-EpCam antibody (Clone 1B7) was obtained from eBioscience. Anti-Axl antibody D was a kind gift obtained from Genmab and produced as described in [3].

Generation and purification of Fab fragments

Crude papain (Sigma, Papain from papaya latex, P3375) was dissolved in papain buffer (40 mM sodium phosphate pH 6.5, 100 mM NaCl) to a concentration of 1 mg/mL. The solution forms a suspension and hence it was filtered through a 0.2 µm filter. Following components were mixed: 120 µL Cetuximab (5 mg/mL), 60 µL papain (1 mg/mL), 60 µL 10x activation buffer (10 mM EDTA, 50 mM Cysteine pH 5.5), 300 µL papain buffer. The reaction was incubated at 37 °C for 2 hours. After 2 hours, 60 µL 1 M Tris pH 8.0 and 26.4 µL 25x complete protease inhibitor (Roche) was added to neutralize the pH. 500 µL of the digestion reaction mixture was loaded on a 500 µL M-8his affinity resin column (See M-8his functionalization of sulfolink resin) equilibrated in wash buffer (20 mM Tris pH 8.0, 100 mM NaCl, 5 mM EDTA) and incubated for 5 minutes. The column was washed with 10 column volumes (= 5 mL) of wash buffer. 300 µL elution buffer (40 mM sodium formate pH 3.0) was added to the column (Pre-elution). 500 µL elution buffer was added to the column and incubated for 5 minutes at room temperature, the flow through was collected and immediately neutralized by the addition of 110 µL 1 M Tris pH 8.0. Another 500 µL elution buffer was added to the column and the flow through was combined with the first elution fraction. The yield was calculated by measuring the A_{280} of the eluate.

Generation and purification of (Fab')$_2$ fragments

Crude pepsin (Sigma, Pepsin from porcine gastric mucosa, P7000) was dissolved in pepsin buffer (40 mM sodium formate pH 2.0) to a concentration of 10 mg/mL. The solution was filtered. Following components were mixed: 120 µL Cetuximab (5 mg/mL), 60 µL pepsin (10 mg/mL), 360 µL pepsin buffer. The reaction was incubated at 37 °C for 1 hours. After 1 hour, 60 µL 1 M Tris pH 8.0 and 26.4 µL 25x protease inhibitor (Roche) was added to neutralize the pH. 500 µL of the digestion reaction mixture was loaded on a 500 µL M-8his resin column equilibrated in wash buffer (20 mM Tris pH 8.0, 100 mM NaCl, 5 mM EDTA) and incubated for 5 minutes. The column was washed with 10 column volumes (= 5 mL) of wash buffer. 300 µL elution buffer (40 mM sodium formate pH 3.0) was added to the column (Pre-elution). 500 µL elution buffer was added to the column and incubated for 5 minutes at room temperature, the flow through was collected and immediately neutralized by the addition of 110 µL 1 M Tris pH 8.0. Another 500 µL elution buffer was added to the column and the flow through was combined with the first elution fraction. The yield was calculated by measuring the A_{280} of the eluate.

Purification of IgG from bacterial lysate

5 mL from an overnight culture of *E. coli* Dh5α cells in LB medium was centrifuged. The pellet was resuspended in 400 µL bugbuster (Novagen) supplemented with benzonase. Cells were lysed for 30 minutes at room temperature. The lysate was cleared by centrifugation. To 90 µL of the lysate cetuximab was added to a final concentration of 1 µM and the volume was adjusted to 100 µL with wash buffer. 50 µL was loaded on 16 µL M-8his affinity resin and purified using the spin column purification procedure (see repeated M-8his affinity resin purification).

Purification of trastuzumab scFv using periplasmic extraction

A freshly transformed Bl21 DE3 colony containing the pET28a trastuzumab ScFv plasmid was grown in 5 mL LB + 50 µg/mL kanamycin overnight at 37 °C. 250 µL of the overnight culture was added to an erlenmeyer flask containig 25 mL LB medium + 50 µg/mL kanamycin. Cells were grown to an O.D. of 0.5 at 37 °C. IPTG was added to a final concentration of 200 µM. Protein expression was induced for 16h at 25 °C. Cells were harvested by centrifugation (2000 xg, 10 minutes) and the pellet was resuspended in hypertonic buffer (200 mM Tris pH 8.0, 20% w/v sucrose, 0.5 mM EDTA, 10 mL/g pellet) + 1x protease inhibitor (Roche) and incubated on ice for 30 minutes. Cells were centrifuged (2000 xg, 10 minutes) and the supernatant was collected. The pellet was resuspended in hypotonic buffer (200 mM Tris pH 8.0, 15 mM $MgSO_4$, 10 mL/g pellet) protease inhibitor (Roche) and incubated on ice for 30 minutes. Cells were centrifuged (2000 xg, 10 minutes.) and the supernatant was combined with the hypertonic supernatant. The combined supernatant was filtered and incubated with M-8his affinity resin (500 µL resin) for 5 minutes at room temperature. The column was washed with 5 mL of wash buffer (20 mM Tris pH 8.0, 100 mM NaCl, 5 mM EDTA). The scFv was eluted from the column with 1 mL elution buffer (40 mM sodium formate pH 3.0). The pH in the eluate was immediately neutralized by the addition of 100 µL 1 M Tris pH 8.0. The purified scFv was concentrated using vivaspin concentrators.

Purification of Adalimumab scFv's and CR6261 -mNeonGreen scFv's

Adalimumab V_HV_L, V_LV_H, CR6261 V_HV_L mNG and CR6261 V_LV_H mNeonGreen were not purified via periplasmic extraction, but according to a modified procedure to improve the yield. A freshly transformed Bl21 DE3 colony containing the pET24a scFv plasmid was grown in 5 mL LB + 50 µg/mL kanamycin. The overnight culture was used to inoculate 1 L of 2YT medium and incubated at 37 °C until the $O.D._{600}$ reached ~0.5. Expression was induced by the addition of Isopropyl β-D-1-thiogalactopyramoside (IPTG) to a final concentration of 100 µM. After overnight expression at 20 °C the cells were harvested by centrifugation and subsequently lysed by Bugbuster protein extraction reagent (Novagen) supplemented with benzonase for 1 hour at room temperature. The lysate was cleared by centrifugation and subsequently filtered through a 0.2 µm filter and applied to a column containing His-bind resin. The column was washed with 10 CV of buffer A (PBS + 370 mM NaCl + 10% v/v glycerol + 20 mM imidazole) and subsequently eluted with elution buffer (basic buffer + 230 mM imidazole). The eluate was applied on a 0.5 mL M-8his affinity resin column. The column was washed with 5 mL of wash buffer (20 mM Tris pH 8.0, 100 mM NaCl, 5 mM EDTA). The scFv was eluted from the column with 3 mL elution buffer (40 mM sodium formate pH 3.0). The pH in the eluate was immediately neutralized by the addition of 300 µL 1 M Tris pH 8.0. The eluate was loaded on a PD-10 column equilibrated in PBS. Purified protein was flash frozen in liquid nitrogen and stored at -80 °C until further use.

Adalimumab HL and adalimumab LH scFv were labeled with Cy3-NHS ester (lumiprobe) according to the manufacturer's instructions and subsequently purified with M-8his affinity resin to remove unreacted dye and scFv that cannot bind anymore to M-8his affinity resin as a result of Cy3 labeling.

Infliximab photoconjugation and subsequent purification

Protein Gx-Large BiT and protein Gx-small BiT were expressed and purified according to a reported procedure[1]. A photoconjugation reaction was set up consisting of 12 µM Gx-LB or Gx-SB[1] and 1.5 µM infliximab in PBS was prepared, 100 µL final volume. Photoconjugation was performed for 10 minutes using a 2 W 365 nm UV lamp (M365LP1, Thorlabs) at 50% power output. 50 µL of the reaction was added to 25 µL of M-8his affinity resin in micro-spin columns (Pierce) and incubated for 10 minutes. The micro-spin columns were centrifuged and the resin was resuspended in 150 µL wash buffer (20

mM Tris pH 8.0, 100 mM NaCl, 5 mM EDTA). The micro-spin columns were centrifuged and the affinity resin was resuspended in 25 µL (protein Gx Small BiT photoconjugation) or 40 µL (protein Gx Large BiT photoconjugation) 40 mM sodium formate pH 4.0 and incubated for 5 minutes at room temperature. The micro-spin columns were centrifuged and the pH of the eluate was immediately neutralized by the addition of 10 µL 1 M Tris pH 8.0. The affinity resin was resuspended a second time with 25 µL or 40 µL 40 mM sodium formate pH 4.0, incubated for 5 minutes and centrifuged. The eluate was combined with the first eluate and the protein concentration was measured using Bradford reagent using a dilution series of infliximab as a standard.

TNFα sensing using purified and unpurified photoconjugation products

A stock solution of 2.5 nM infliximab-protein Gx Large BiT, 2.5 nM infliximab-protein Gx Small BiT was prepared in PBS + 1 mg/mL BSA, using either purified or unpurified Photoconjugation products. A 2-fold dilution series of TNFα was prepared starting from 100 nM TNFα in PBS + 1 mg/mL BSA. In triplicate, 8 µL of the photoconjugation stock solution was mixed with 10 µL of TNFα solution and incubated for 30 minutes at room temperature in a white 384-wells plate. After 30 minutes 2 µL of a 1:100 nanoglo solution in PBS + 1 mg/mL BSA was added to each well and the luminescence signal was recorded on a Tecan Spark 10 M plate reader.

Determining the affinity of scFv for protein M WT and M-8his using BRET

A 2-fold dilution series of Cy3-labeled adalimumab scFv or CR6261 scFv mNeonGreen was prepared in PBS + 1 mg/mL BSA. 10 µL of scFv was mixed with 8 µL of 125 pM (12.5 pM for CR6261 scFv mNeonGreen) NanoLuc-protein M fusion protein in PBS and added to a white 384-wells plate and incubated for 1 h at room temperature. 2 µL of a 1:100 dilution of nanoglo(Promega) in PBS + 1 mg/mL BSA was added to each well and the luminescence spectrum was recorded on a Tecan Spark 10 M plate reader. Experiments were performed in triplicate. K_D values were obtained by fitting the emission ratio as function of scFv concentration using equation S2.

$$R = R_o - (R_o - R_{max})[scFv]/(K_D + [scFv]) \qquad \text{Equation S1}$$

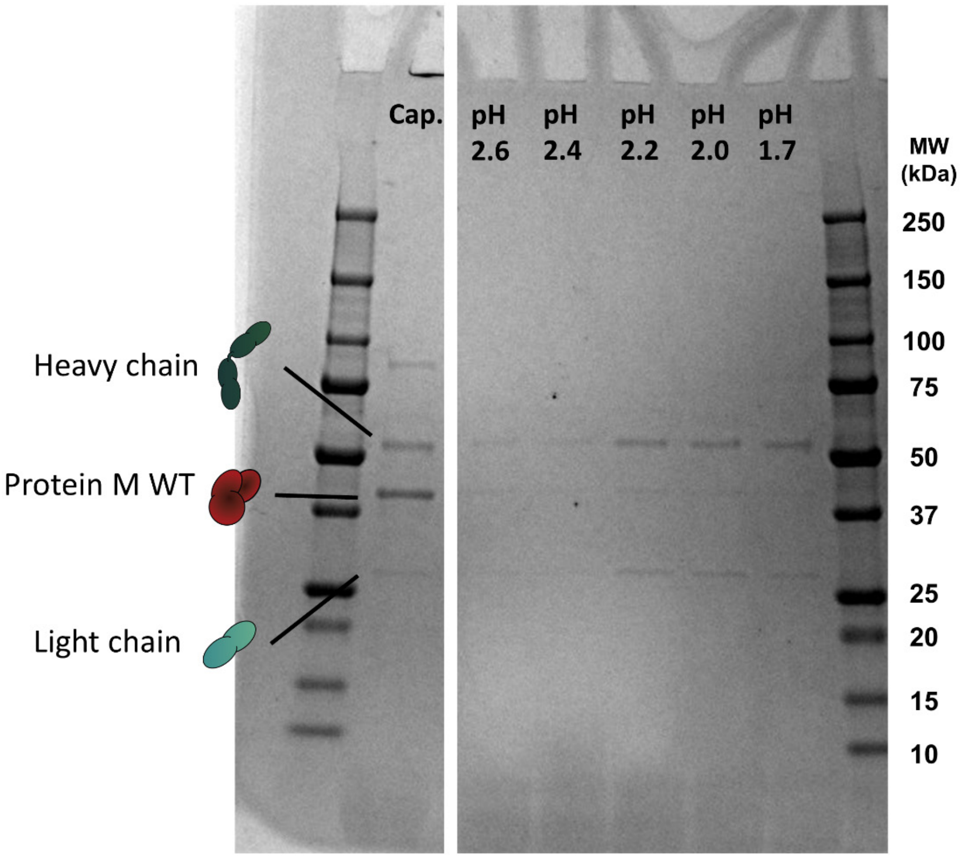

Figure S1. Reducing SDS-PAGE gel of cetuximab elution from protein-M-WT-functionalized superparamagnetic beads. 0.6 mg of protein-M-WT-functionalized superparamagnetic beads was resuspended in 75 µL 0.89 µM cetuximab in PBS and incubated for 30 minutes at RT. After washing the beads were resuspended in 45 µL 50 mM glycine at the indicated pH at 55 °C for 2 min.

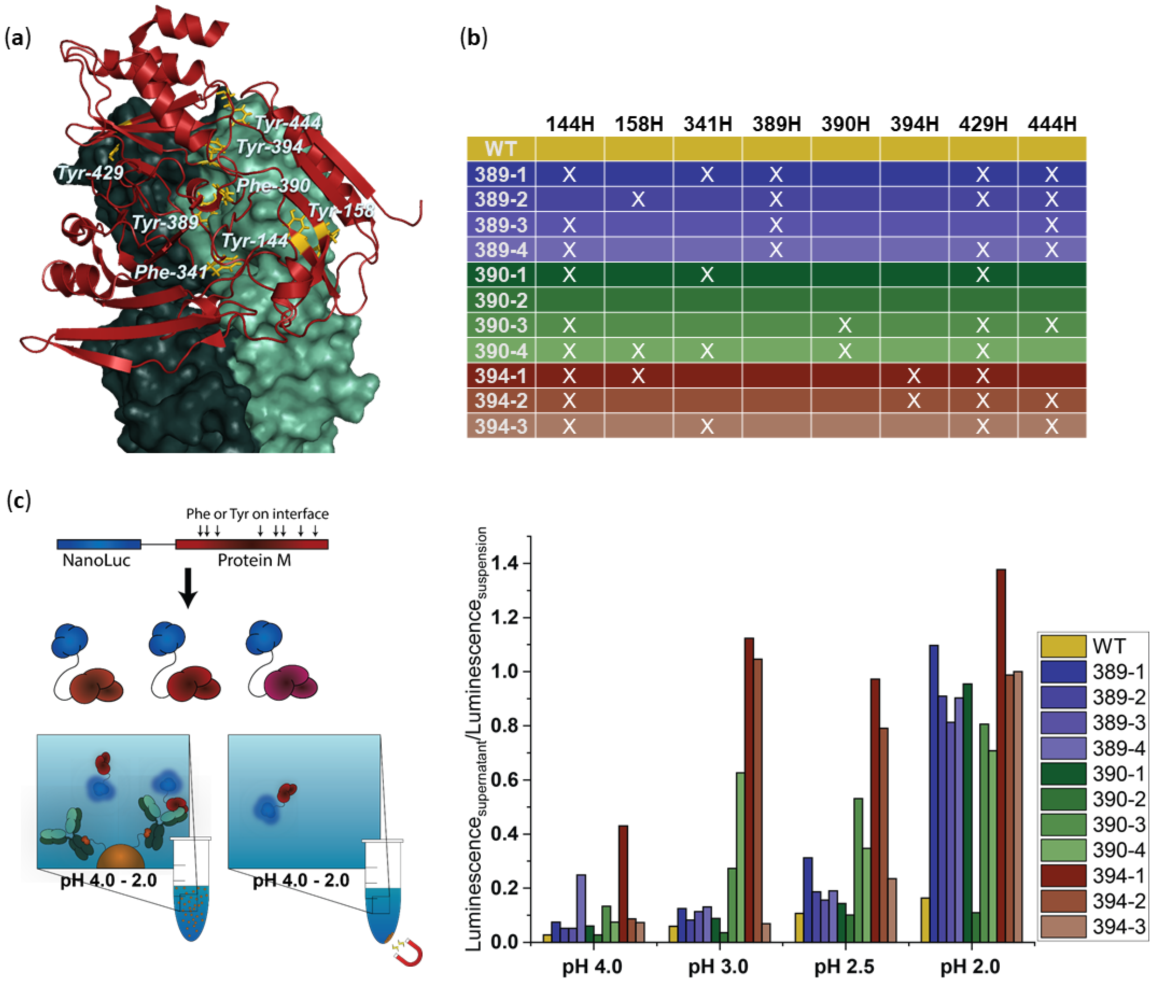

	144H	158H	341H	389H	390H	394H	429H	444H
WT								
389-1	X		X	X			X	X
389-2		X		X			X	X
389-3	X			X				X
389-4	X			X			X	X
390-1	X		X				X	
390-2								
390-3	X				X		X	X
390-4	X	X	X		X		X	
394-1	X	X				X	X	
394-2	X					X	X	X
394-3	X		X				X	X

Figure S2. (**a**) Structural representation of Protein M in complex with Fab fragment (PDB: 4nzr[4]). Dark green represents the heavy chain, light green the light chain, red represents protein M. (**b**) Histidine substitutions of the 4 members of each library that were screened. 394-4 is omitted since this mutant contained a frameshift, producing a non-functional protein. (**c**) Screening results of 4 members of each library.

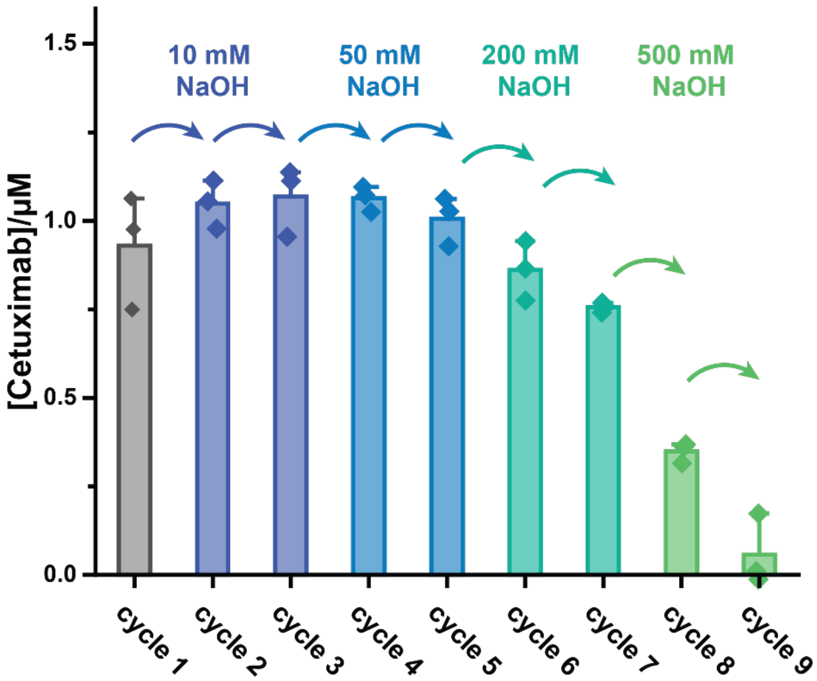

Figure S3. Reusability of M-8his affinity resin after regeneration with sodium hydroxide. For 8 cycles, an excess (100 µL, 2.5 µM) cetuximab was incubated with M-8his affinity resin. The bound fraction was eluted with 100 µL sodium formate pH 3.0, followed by a 5 min. regeneration step with 10, 50, 200 or 500 mM sodium hydroxide. The cetuximab concentration in each elution step was determined by a Bradford assay.

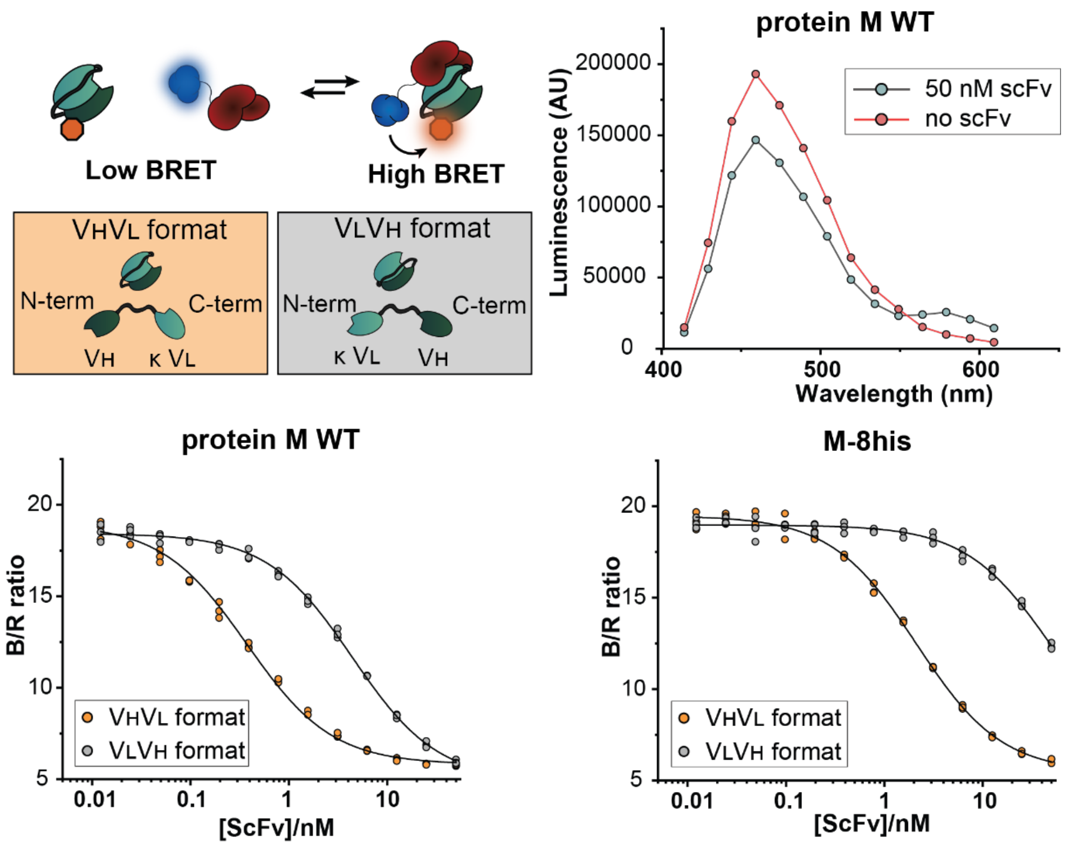

Figure S4. Determining the affinity of NanoLuc-protein M WT and M-8his for V_H-V_L- and V_L-V_H-adalimumab scFv. 50 pM NanoLuc-protein M was incubated with 0-50 nM Cy3-functionalized adalimumab scFv for 1h. After the addition of NanoGlo, the luminescence spectrum was recorded. The Blue/Red (B/R) ratio was calculated by dividing the luminescence signal at 458 nm by the signal at 578 nm. Lines represent best fits to equation S2 yielding the K_D values listed in Table s1.

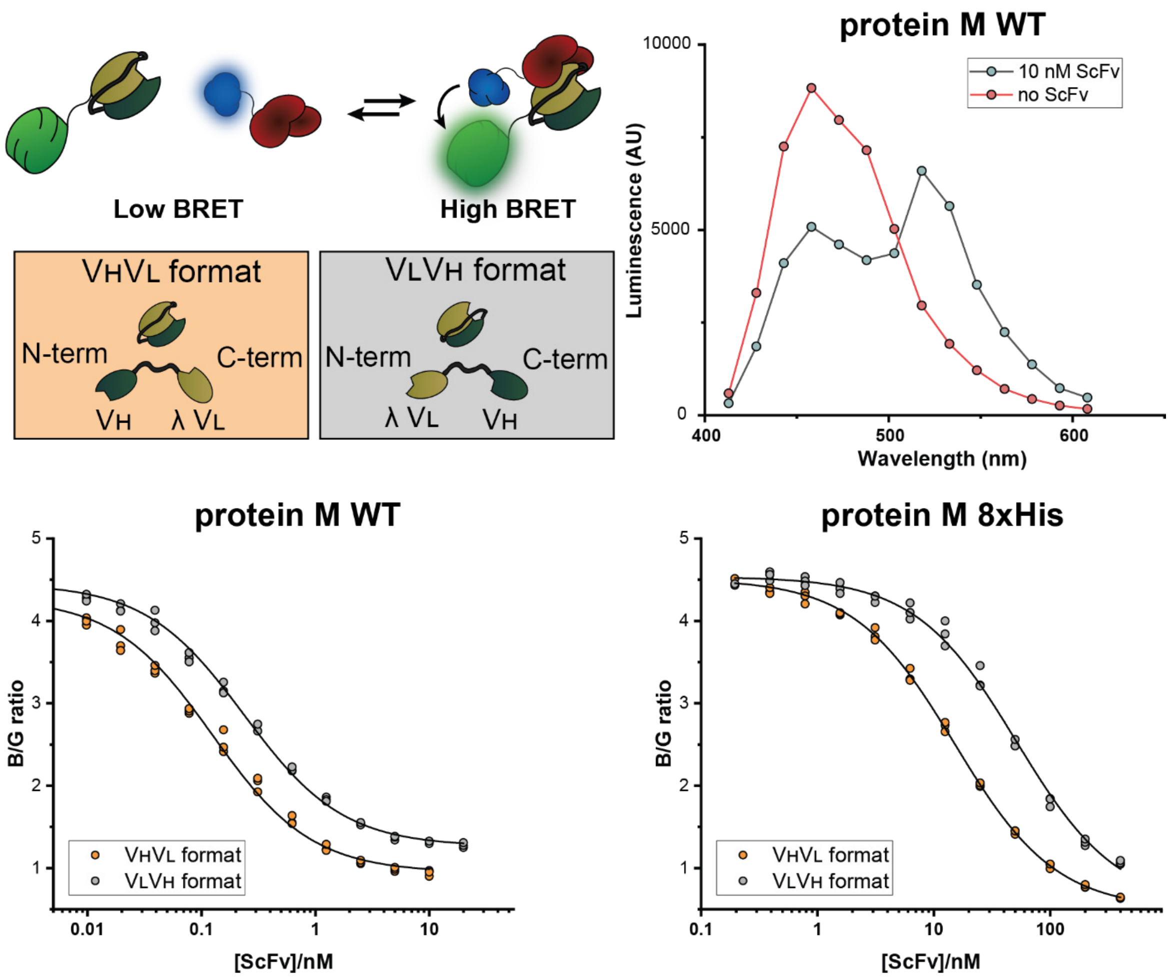

Figure S5. Determining the affinity of NanoLuc-protein M WT and M-8his for V_H-V_L- and V_L-V_H-CR6261 scFv. 5 pM NanoLuc-protein M was incubated with 0-20 nM (protein M WT) or 0-400 nM (M-8his) CR6261 mNeonGreen for 1h. After the addition of NanoGlo, the luminescence spectrum was recorded. The Blue/Green (B/G) ratio was calculated by dividing the luminescence signal at 458 nm by the signal at 533 nm. Lines represent best fits to equation S2 yielding the K_D values listed in Table s1.

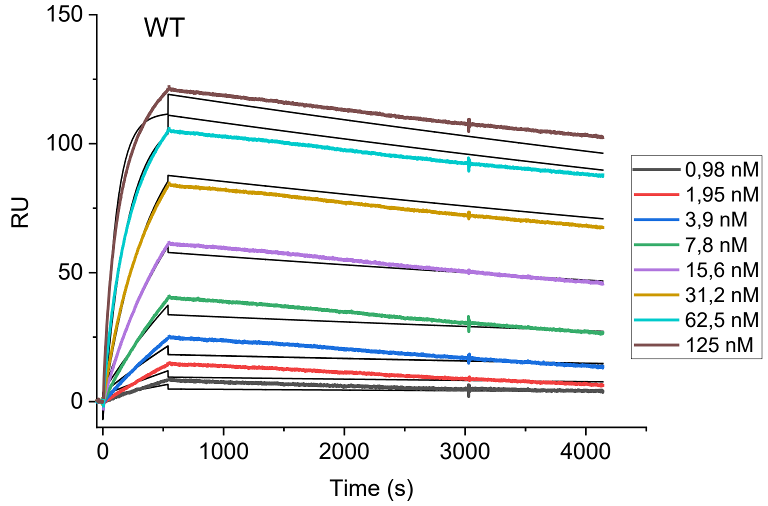
WT
RU
Time (s)
0,98 nM
1,95 nM
3,9 nM
7,8 nM
15,6 nM
31,2 nM
62,5 nM
125 nM

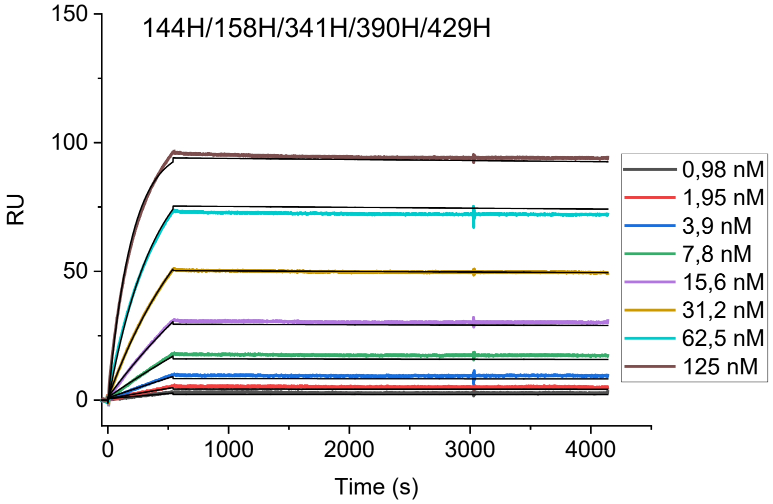
144H/158H/341H/390H/429H
RU
Time (s)
0,98 nM
1,95 nM
3,9 nM
7,8 nM
15,6 nM
31,2 nM
62,5 nM
125 nM

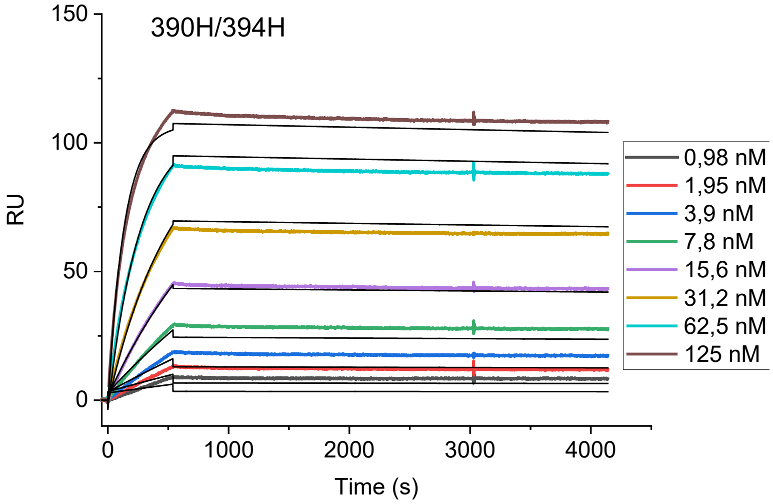
390H/394H
RU
Time (s)
0,98 nM
1,95 nM
3,9 nM
7,8 nM
15,6 nM
31,2 nM
62,5 nM
125 nM

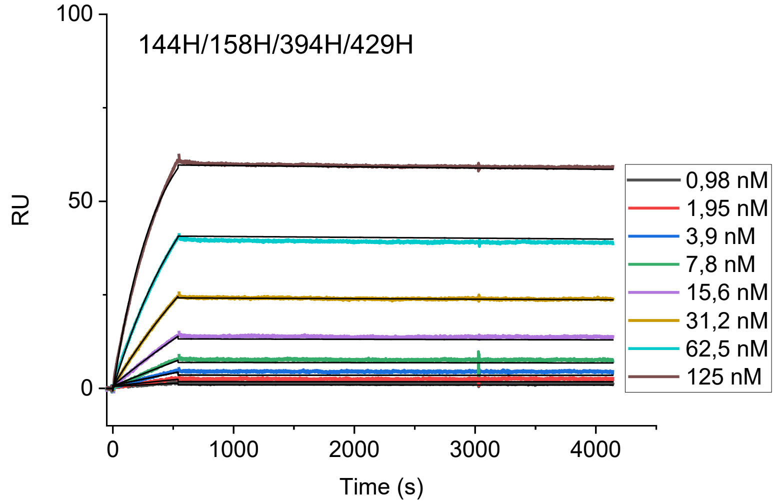

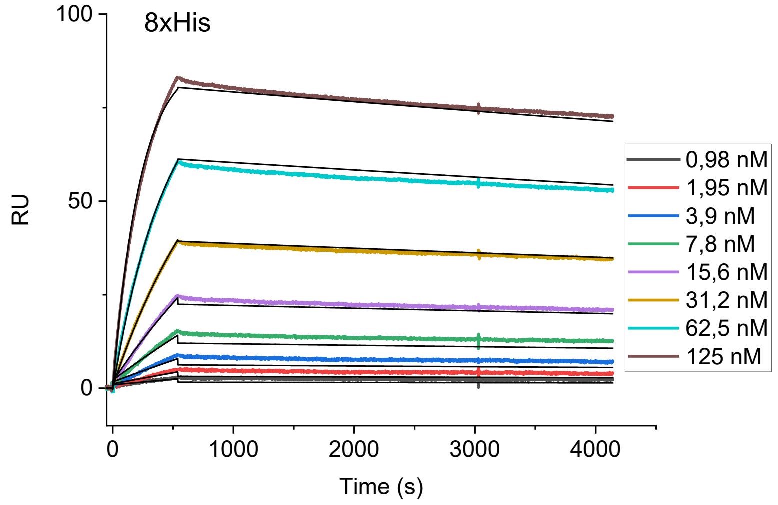

Figure S6. SPR measurements of binding and dissociation of protein M wild-type, protein M F390H/Y394H, protein M Y144H/Y158H/F341H/F390H/Y429H, protein M Y144H/Y158H/Y394H/Y429H and protein M 8×His to immobilized cetuximab Fab at pH 7.5. The blank-subtracted sensor grams are plotted as colorful lines and the 1:1 binding fitted curves are plotted as black lines. The calculated parameters are listed in Table S2.

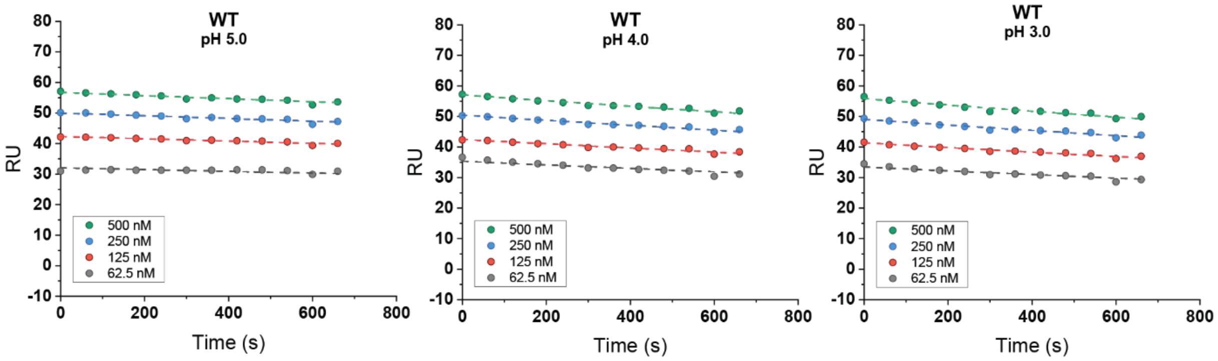

Figure S7. Dissociation of protein M WT from immobilized cetuximab Fab at pH 5.0, 4.0 and 3.0. Indicated are datapoints and fit to Equation S1.

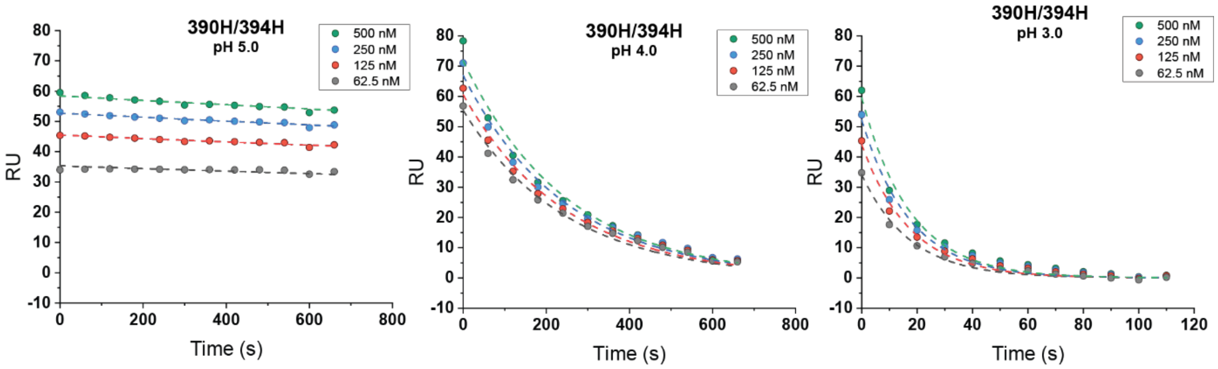

Figure S8. Dissociation of protein M 390H/394H from immobilized cetuximab Fab at pH 5.0, 4.0 and 3.0. Indicated are datapoints and fit to Equation S1.

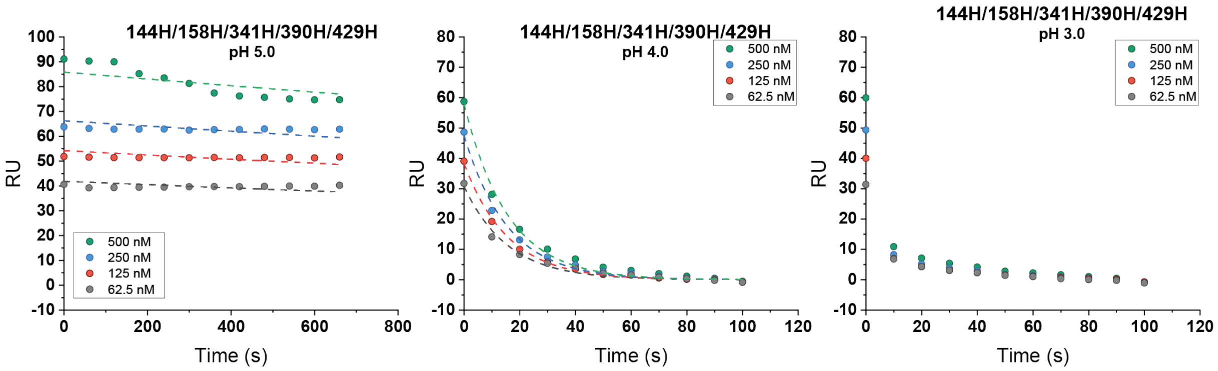

Figure S9. Dissociation of protein M 144H/158H/341H/390H/429H from immobilized cetuximab Fab at pH 5.0, 4.0 and 3.0. Indicated are datapoints and fit to Equation S1.

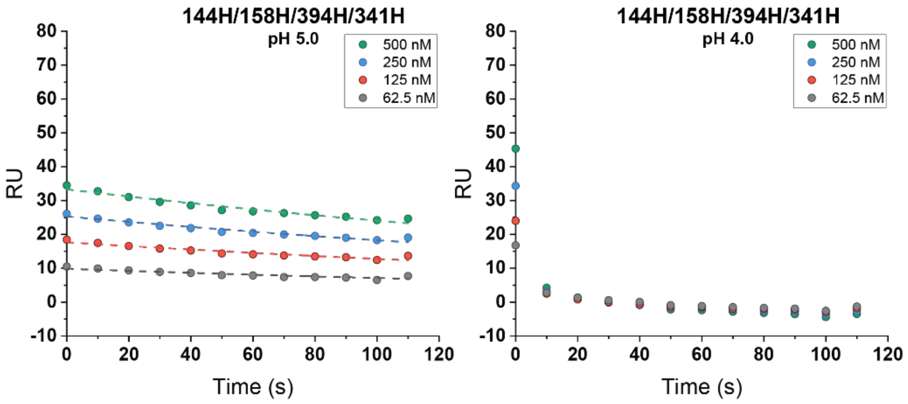

Figure S10. Dissociation of protein M 144H/158H/341H/394H from immobilized cetuximab Fab at pH 5.0 and 4.0. Indicated are datapoints and fit to Equation S1.

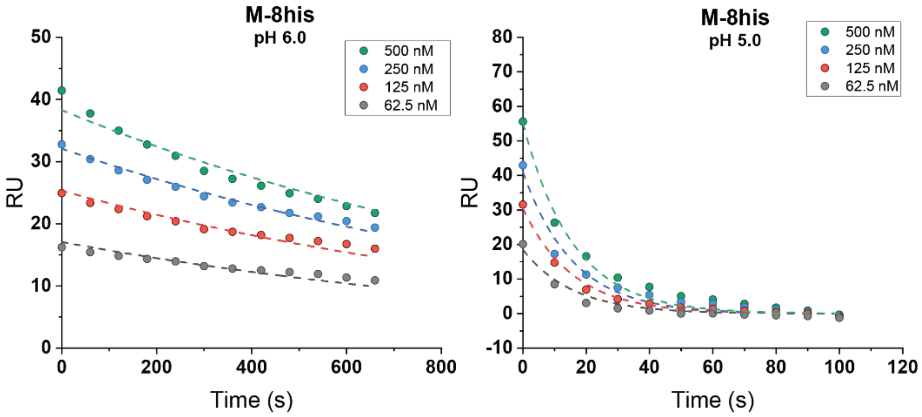

Figure S11. Dissociation of M-8his from immobilized cetuximab Fab at pH 6.0 and 5.0. Indicated are datapoints and fit to Equation S1.

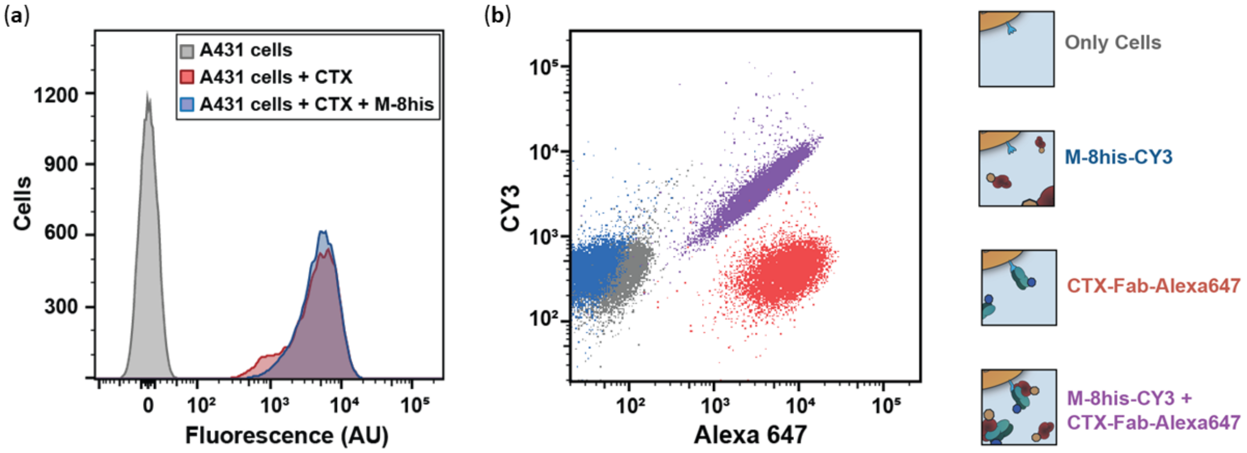

Figure S12. (**a**) FACS analysis of A431 cells labeled with 1 nM cetuximab and 1 nM M-8his-complexed cetuximab at pH 7.4. (**b**) FACS scatter plot of A431 cells labeled with 1 nM alexa647-cetuximab Fab, 5 nM Cy3-M-8his, or 1 nM Cy3-M-8his-complexed with Alexa647-cetuximab Fab at pH 7.4.

```
M  G  S  S  H  H  H  H  H  H  S  S  G  L  V  P  R  G  S  H
ATGGGCAGCAGCCATCATCATCATCATCACAGCAGCGGCCTGGTGCCGCGCGGCAGCCAT
M  S  L  S  L  N  D  G  S  Y  Q  S  E  I  D  L  S  G  G  A
ATGTCGCTTAGTCTTAATGACGGCTCGTATCAAAGTGAGATCGATCTTTCCGGAGGTGCG
N  F  R  E  K  F  R  N  F  A  N  E  L  S  E  A  I  T  N  S
AACTTCCGGGAGAAGTTTCGTAATTTCGCTAATGAGCTTTCAGAGGCTATAACCAACAGT
P  K  G  L  D  R  P  V  P  K  T  E  I  S  G  L  I  K  T  G
CCGAAGGGACTGGACAGACCTGTCCCAAAGACAGAAATAAGCGGCCTGATTAAAACGGGA
D  N  F  I  T  P  S  F  K  A  G  Y  Y  D  H  V  A  S  D  G
GATAACTTCATTACACCGAGCTTCAAGGCGGGATACTATGATCACGTCGCATCTGACGGT
S  L  L  S  Y  Y  Q  S  T  E  Y  F  N  N  R  V  L  M  P  I
TCCTTACTTTCGTATTACCAAAGTACGGAGTACTTCAATAACCGCGTGCTGATGCCCATA
L  Q  T  T  N  G  T  L  M  A  N  N  R  G  Y  D  D  V  F  R
TTGCAAACCACGAATGGGACATTAATGGCGAATAACCGCGGCTACGACGATGTATTCCGG
Q  V  P  S  F  S  G  W  S  N  T  K  A  T  T  V  S  T  S  N
CAGGTCCCATCGTTCTCGGGCTGGAGCAATACTAAGGCCACCACTGTATCAACTTCGAAT
N  L  T  Y  D  K  W  T  Y  F  A  A  K  G  S  P  L  Y  D  S
AATCTTACCTATGACAAGTGGACGTACTTCGCCGCAAAAGGCTCACCTCTGTATGATAGC
Y  P  N  H  F  F  E  D  V  K  T  L  A  I  D  A  K  D  I  S
TACCCTAATCATTTTTTTGAAGATGTCAAAACATTAGCTATCGATGCTAAAGATATCTCG
A  L  K  T  T  I  D  S  E  K  P  T  Y  L  I  I  R  G  L  S
GCCTTAAAAACTACTATCGATTCGGAGAAACCCACATATTTGATAATACGCGGACTGTCT
G  N  G  S  Q  L  N  E  L  Q  L  P  E  S  V  K  K  V  S  L
GGCAATGGCTCGCAGTTGAATGAGCTGCAATTGCCCGAGTCTGTCAAAAAAGTCTCCCTT
Y  G  D  Y  T  G  V  N  V  A  K  Q  I  F  A  N  V  V  E  L
TATGGAGATTATACCGGGGTTAATGTCGCCAAACAAATTTTTGCGAATGTAGTTGAACTT
E  F  Y  S  T  S  K  A  N  S  F  G  F  N  P  L  V  L  G  S
GAATTTTACAGCACAAGCAAGGCAAATAGTTTCGGATTCAACCCGTTGGTATTAGGATCA
K  T  N  V  I  Y  D  L  F  A  S  K  P  F  T  H  I  D  L  T
AAGACCAATGTAATTTATGACTTATTCGCGTCAAAACCTTTTACTCACATAGATTTGACC
Q  V  T  L  Q  N  S  D  N  S  A  I  D  A  N  K  L  K  Q  A
CAGGTAACCCTGCAAAACTCCGACAATTCCGCAATCGATGCTAACAAACTGAAACAAGCA
V  G  D  I  Y  N  Y  R  R  F  E  R  Q  F  Q  G  Y  F  A  G
GTGGGCGACATCTATAACTATAGAAGATTTGAACGCCAATTTCAGGGTTACTTTGCAGGA
G  Y  I  D  K  Y  L  V  K  N  V  N  T  N  K  D  S  D  D  D
GGCTATATCGACAAATACCTTGTGAAAAATGTCAACACTAACAAGGACTCTGACGACGAT
L  V  Y  R  S  L  K  E  L  N  L  H  L  E  E  A  Y  R  E  G
TTAGTATACCGGAGTTTGAAAGAGCTTAATCTGCATTTGGAAGAGGCCTATCGGGAAGGC
D  N  T  Y  Y  R  V  N  E  N  Y  Y  P  G  A  S  I  Y  E  N
GATAATACATATTACCGCGTAAACGAGAACTACTATCCGGGTGCATCAATCTACGAAAAC
E  R  A  S  R  D  S  E  F  Q  N  E  I  L  K  R  G  G  S  G
GAGCGGGCGAGCCGCGATTCCGAATTTCAAAACGAGATCCTTAAACGTGGTGGAAGTGGG
G  S  W  S  H  P  Q  F  E  K  C  *
GGCAGCTGGTCCCATCCGCAGTTCGAGAAATGCTGA
```

His tag

Protein M TD

Residues mutated to His in M-8his

Strep tag

Figure S13. DNA and corresponding amino acid sequence of protein M

```
M  G  S  S  H  H  H  H  H  H  S  S  G  L  V  P  R  G  S  H
ATGGGCAGCAGCCATCATCATCATCATCACAGCAGCGGCCTGGTGCCGCGCGGCAGCCAT
M  K  Y  L  L  P  T  A  A  A  G  L  L  L  L  A  A  Q  P  A
ATGAAATATCTGCTTCCAACCGCAGCAGCCGGCTTACTGTTGCTGGCTGCGCAGCCGGCG
M  A  E  V  Q  L  V  E  S  G  G  G  L  V  Q  P  G  G  S  L
ATGGCCGAGGTGCAGTTGGTAGAAAGCGGTGGCGGTCTGGTGCAGCCAGGTGGGTCACTG
R  L  S  C  A  A  S  G  F  N  I  K  D  T  Y  I  H  W  V  R
CGTTTGAGTTGTGCCGCGTCGGGTTTCAACATCAAGGATACCTACATTCATTGGGTTCGT
Q  A  P  G  K  G  L  E  W  V  A  R  I  Y  P  T  N  G  Y  T
CAAGCACCGGGTAAAGGACTGGAATGGGTTGCCCGCATTTATCCAACGAACGGCTACACA
R  Y  A  D  S  V  K  G  R  F  T  I  S  A  D  T  S  K  N  T
AGATACGCGGATTCGGTGAAAGGCCGATTCACGATCTCCGCCGATACGAGCAAGAATACT
A  Y  L  Q  M  N  S  L  R  A  E  D  T  A  V  Y  Y  C  S  R
GCATATTTACAAATGAACTCACTGCGCGCGGAAGATACGGCGGTGTATTATTGCTCGCGT
W  G  G  D  G  F  Y  A  M  D  Y  W  G  Q  G  T  L  V  T  V
TGGGGTGGCGACGGGTTTTACGCTATGGACTATTGGGGCCAGGGAACTTTAGTGACGGTT
S  S  G  G  G  G  S  G  G  G  G  S  G  G  G  G  S  D  I  Q
TCGAGCGGCGGTGGCGGTAGTGGAGGTGGCGGGTCAGGTGGGGGTGGCTCTGATATTCAA
M  T  Q  S  P  S  S  L  S  A  S  V  G  D  R  V  T  I  T  C
ATGACCCAGTCCCCGTCATCCCTGTCTGCGTCCGTGGGTGATCGCGTTACCATCACCTGC
R  A  S  Q  D  V  N  T  A  V  A  W  Y  Q  Q  K  P  G  K  A
CGGGCTAGTCAAGACGTCAATACCGCAGTTGCCTGGTATCAACAGAAACCGGGCAAAGCA
P  K  L  L  I  Y  S  A  S  F  L  Y  S  G  V  P  S  R  F  S
CCGAAACTCCTGATATATTCTGCTTCTTTTCTATACAGCGGAGTGCCGAGCCGTTTTTCA
G  S  R  S  G  T  D  F  T  L  T  I  S  S  L  Q  P  E  D  F
GGGAGTCGCAGCGGTACCGATTTCACGCTTACCATTAGTAGCCTGCAGCCGGAGGACTTT
A  T  Y  Y  C  Q  Q  H  Y  T  T  P  P  T  F  G  Q  G  T  K
GCGACATATTACTGTCAGCAGCACTATACAACCCCTCCTACTTTTGGCCAGGGCACTAAA
V  E  I  K  G  S  G  G  G  W  *
GTAGAAATTAAAGGCTCCGGCGGCGGCTGGTGA
```

PelB sequence

Heavy chain

Light chain

His tag

Figure S14. DNA and corresponding amino acid sequence of trastuzumab scFv.

```
M  K  Y  L  L  P  T  A  A  A  G  L  L  L  L  A  A  Q  P  A
ATGAAGTACCTGCTGCCGACCGCAGCAGCGGGTTTATTGCTGCTGGCAGCACAACCAGCT
M  A  E  V  Q  L  V  E  S  G  A  E  V  K  K  P  G  S  S  V
ATGGCCGAAGTGCAACTTGTAGAATCCGGCGCTGAAGTCAAAAAGCCGGGGTCCTCAGTA
K  V  S  C  K  A  S  G  G  P  F  R  S  Y  A  I  S  W  V  R
AAGGTAAGCTGCAAAGCCTCAGGTGGACCTTTCCGCAGCTATGCGATTAGTTGGGTTCGC
Q  A  P  G  Q  G  P  E  W  M  G  G  I  I  P  I  F  G  T  T
CAAGCGCCAGGACAGGGCCCGGAATGGATGGGGGGTATTATTCCCATTTTCGGGACAACG
K  Y  A  P  K  F  Q  G  R  V  T  I  T  A  D  D  F  A  G  T
AAATATGCACCAAAATTCCAAGGTCGCGTCACGATTACTGCCGATGACTTTGCCGGCACC
V  Y  M  E  L  S  S  L  R  S  E  D  T  A  M  Y  Y  C  A  K
GTGTATATGGAGTTATCGTCCCTTCGCTCTGAAGACACTGCTATGTATTACTGTGCCAAA
H  M  G  Y  Q  V  R  E  T  M  D  V  W  G  K  G  T  T  V  T
CACATGGGCTACCAGGTTCGTGAGACCATGGACGTTTGGGGGAAAGGGACCACTGTGACC
V  S  S  G  G  G  G  S  G  G  G  G  S  G  G  G  G  S  Q  S
GTATCGTCAGGAGGGGGCGGTAGTGGCGGCGGAGGATCAGGAGGCGGAGGATCCCAGAGC
V  L  T  Q  P  P  S  V  S  A  A  P  G  Q  K  V  T  I  S  C
GTTTTAACACAACCACCATCGGTCAGTGCAGCTCCGGGCCAAAAAGTAACTATCTCTTGC
S  G  S  S  S  N  I  G  N  D  Y  V  S  W  Y  Q  Q  L  P  G
TCGGGTTCCTCTTCCAATATTGGGAATGACTACGTTTCGTGGTATCAACAGTTACCGGGC
T  A  P  K  L  L  I  Y  D  N  N  K  R  P  S  G  I  P  D  R
ACAGCTCCCAAGTTACTTATCTACGACAATAACAAACGTCCGTCTGGGATTCCGGATCGT
F  S  G  S  K  S  G  T  S  A  T  L  G  I  T  G  L  Q  T  G
TTTTCAGGCAGTAAATCGGGGACCTCTGCCACTTTAGGAATCACGGGTTTGCAAACGGGG
D  E  A  N  Y  Y  C  A  T  W  D  R  R  P  T  A  Y  V  V  F
GATGAGGCGAATTACTATTGTGCGACCTGGGATCGTCGCCCTACAGCATACGTTGTATTT
G  G  G  T  K  L  T  V  L  G  T  S  G  S  H  M  V  S  K  G
GGGGGTGGCACCAAGTTGACTGTGCTTGGTACCTCAGGCAGCCATATGGTAAGTAAAGGT
E  E  D  N  M  A  S  L  P  A  T  H  E  L  H  I  F  G  S  I
GAAGAAGACAATATGGCTTCTCTGCCTGCCACACATGAGCTTCATATTTTTGGGAGCATA
N  G  V  D  F  D  M  V  G  Q  G  T  G  N  P  N  D  G  Y  E
AACGGAGTGGATTTCGACATGGTAGGTCAGGGTACGGGGAACCCTAACGATGGATATGAG
E  L  N  L  K  S  T  K  G  D  L  Q  F  S  P  W  I  L  V  P
GAGTTGAATCTTAAAAGCACAAAGGGTGATCTGCAGTTCTCGCCCTGGATCCTGGTGCCG
H  I  G  Y  G  F  H  Q  Y  L  P  Y  P  D  G  M  S  P  F  Q
CATATAGGTTATGGTTTCCATCAGTATCTTCCATACCCGGATGGCATGAGCCCTTTTCAG
A  A  M  V  D  G  S  G  Y  Q  V  H  R  T  M  Q  F  E  D  G
GCCGCAATGGTAGATGGCTCAGGATATCAAGTGCATCGGACCATGCAGTTTGAAGATGGG
A  S  L  T  V  N  Y  R  Y  T  Y  E  G  S  H  I  K  G  E  A
GCGTCTTTGACGGTAAATTACAGGTACACCTATGAGGGTAGCCATATAAAGGGAGAAGCG
Q  V  K  G  T  G  F  P  A  D  G  P  V  M  T  N  S  L  T  A
CAGGTGAAGGGAACTGGATTCCCAGCGGATGGCCCAGTCATGACAAACAGCCTCACCGCT
A  D  W  C  R  S  K  K  T  Y  P  N  D  K  T  I  I  S  T  F
GCTGATTGGTGCCGATCCAAGAAAACGTATCCAAACGATAAAACTATCATTTCTACTTTT
K  W  S  Y  T  T  G  N  G  K  R  Y  R  S  T  A  R  T  T  Y
AAGTGGTCCTATACAACAGGAAACGGGAAACGCTATCGTTCAACGGCCCGCACGACCTAC
T  F  A  K  P  M  A  A  N  Y  L  K  N  Q  P  M  Y  V  F  R
ACGTTTGCAAAGCCAATGGCTGCGAATTATCTGAAAAACCAGCCGATGTATGTGTTCCGT
K  T  E  L  K  H  S  K  T  E  L  N  F  K  E  W  Q  K  A  F
AAAACCGAACTGAAACATTCTAAAACGGAGCTCAATTTCAAGGAATGGCAGAAGGCATTT
T  G  F  H  H  H  H  H  H  *
ACCGGTTTTCATCATCATCATCATCACTAA
```

PelB sequence

Heavy chain

Light chain

mNeonGreen

His tag

Figure S15. DNA and corresponding amino acid sequence of V_HV_L format CR6261 scFv mNeonGreen fusion protein.

```
M  K  Y  L  L  P  T  A  A  A  G  L  L  L  L  A  A  Q  P  A
ATGAAGTACCTGCTGCCGACCGCAGCAGCGGGTTTATTGCTGCTGGCAGCACAACCAGCT
M  A  Q  S  V  L  T  Q  P  P  S  V  S  A  A  P  G  Q  K  V
ATGGCCCAGAGCGTTTTAACACAACCACCATCGGTCAGTGCAGCTCCGGGCCAAAAAGTA
T  I  S  C  S  G  S  S  S  N  I  G  N  D  Y  V  S  W  Y  Q
ACTATCTCTTGCTCGGGTTCCTCTTCCAATATTGGGAATGACTACGTTTCGTGGTATCAA
Q  L  P  G  T  A  P  K  L  L  I  Y  D  N  N  K  R  P  S  G
CAGTTACCGGGCACAGCTCCCAAGTTACTTATCTACGACAATAACAAACGTCCGTCTGGG
I  P  D  R  F  S  G  S  K  S  G  T  S  A  T  L  G  I  T  G
ATTCCGGATCGTTTTTCAGGCAGTAAATCGGGGACCTCTGCCACTTTAGGAATCACGGGT
L  Q  T  G  D  E  A  N  Y  Y  C  A  T  W  D  R  R  P  T  A
TTGCAAACGGGGGATGAGGCGAATTACTATTGTGCGACCTGGGATCGTCGCCCTACAGCA
Y  V  V  F  G  G  G  T  K  L  T  V  L  S  S  G  G  G  G  S
TACGTTGTATTTGGGGGTGGCACCAAGTTGACTGTGCTTTCGTCAGGAGGGGGCGGTAGT
G  G  G  G  S  G  G  G  G  S  E  V  Q  L  V  E  S  G  A  E
GGCGGCGGAGGATCAGGAGGCGGAGGATCCGAAGTGCAACTTGTAGAATCCGGCGCTGAA
V  K  K  P  G  S  S  V  K  V  S  C  K  A  S  G  G  P  F  R
GTCAAAAAGCCGGGGTCCTCAGTAAAGGTAAGCTGCAAAGCCTCAGGTGGACCTTTCCGC
S  Y  A  I  S  W  V  R  Q  A  P  G  Q  G  P  E  W  M  G  G
AGCTATGCGATTAGTTGGGTTCGCCAAGCGCCAGGACAGGGCCCGGAATGGATGGGGGGT
I  I  P  I  F  G  T  T  K  Y  A  P  K  F  Q  G  R  V  T  I
ATTATTCCCATTTTCGGGACAACGAAATATGCACCAAAATTCCAAGGTCGCGTCACGATT
T  A  D  D  F  A  G  T  V  Y  M  E  L  S  S  L  R  S  E  D
ACTGCCGATGACTTTGCCGGCACCGTGTATATGGAGTTATCGTCCCTTCGCTCTGAAGAC
T  A  M  Y  Y  C  A  K  H  M  G  Y  Q  V  R  E  T  M  D  V
ACTGCTATGTATTACTGTGCCAAACACATGGGCTACCAGGTTCGTGAGACCATGGACGTT
W  G  K  G  T  T  V  T  V  G  T  S  G  S  H  M  V  S  K  G
TGGGGGAAAGGGACCACTGTGACCGTAGGTACCTCAGGCAGCCATATGGTAAGTAAAGGT
E  E  D  N  M  A  S  L  P  A  T  H  E  L  H  I  F  G  S  I
GAAGAAGACAATATGGCTTCTCTGCCTGCCACACATGAGCTTCATATTTTTGGAGCATA
N  G  V  D  F  D  M  V  G  Q  G  T  G  N  P  N  D  G  Y  E
AACGGAGTGGATTTCGACATGGTAGGTCAGGGTACGGGGAACCCTAACGATGGATATGAG
E  L  N  L  K  S  T  K  G  D  L  Q  F  S  P  W  I  L  V  P
GAGTTGAATCTTAAAAGCACAAAGGGTGATCTGCAGTTCTCGCCCTGGATCCTGGTGCCG
H  I  G  Y  G  F  H  Q  Y  L  P  Y  P  D  G  M  S  P  F  Q
CATATAGGTTATGGTTTCCATCAGTATCTTCCATACCCGGATGGCATGAGCCCTTTTCAG
A  A  M  V  D  G  S  G  Y  Q  V  H  R  T  M  Q  F  E  D  G
GCCGCAATGGTAGATGGCTCAGGATATCAAGTGCATCGGACCATGCAGTTTGAAGATGGG
A  S  L  T  V  N  Y  R  Y  T  Y  E  G  S  H  I  K  G  E  A
GCGTCTTTGACGGTAAATTACAGGTACACCTATGAGGGTAGCCATATAAAGGGAGAAGCG
Q  V  K  G  T  G  F  P  A  D  G  P  V  M  T  N  S  L  T  A
CAGGTGAAGGGAACTGGATTCCCAGCGGATGGCCCAGTCATGACAAACAGCCTCACCGCT
A  D  W  C  R  S  K  K  T  Y  P  N  D  K  T  I  I  S  T  F
GCTGATTGGTGCCGATCCAAGAAAACGTATCCAAACGATAAAACTATCATTTCTACTTTT
```

```
K  W  S  Y  T  T  G  N  G  K  R  Y  R  S  T  A  R  T  T  Y
AAGTGGTCCTATACAACAGGAAACGGGAAACGCTATCGTTCAACGGCCCGCACGACCTAC
T  F  A  K  P  M  A  A  N  Y  L  K  N  Q  P  M  Y  V  F  R
ACGTTTGCAAAGCCAATGGCTGCGAATTATCTGAAAAACCAGCCGATGTATGTGTTCCGT
K  T  E  L  K  H  S  K  T  E  L  N  F  K  E  W  Q  K  A  F
AAAACCGAACTGAAACATTCTAAAACGGAGCTCAATTTCAAGGAATGGCAGAAGCATTT
T  G  F  H  H  H  H  H  H  *
ACCGGTTTTCATCATCATCATCATCACTAA
```

PelB sequence

Heavy chain

Light chain

mNeonGreen

His tag

Figure S16. DNA and corresponding amino acid sequence of V_LV_H format CR6261 scFv mNeonGreen fusion protein.

```
M  K  Y  L  L  P  T  A  A  A  G  L  L  L  L  A  A  Q  P  A
ATGAAGTATTTATTGCCAACCGCCGCCGCCGGCCTTCTGTTGTTGGCCGCACAACCCGCA
M  A  E  V  Q  L  V  E  S  G  G  G  L  V  Q  P  G  R  S  L
ATGGCAGAGGTGCAGCTGGTTGAATCTGGCGGTGGTCTTGTTCAACCCGGACGTTCTTTG
R  L  S  C  A  A  S  G  F  T  F  D  D  Y  A  M  H  W  V  R
CGTTTGTCTTGCGCTGCGTCGGGTTTCACCTTCGACGACTATGCCATGCATTGGGTTCGC
Q  A  P  G  K  G  L  E  W  V  S  A  I  T  W  N  S  G  H  I
CAGGCTCCAGGTAAAGGGCTGGAGTGGGTATCCGCGATTACCTGGAACTCTGGTCATATT
D  Y  A  D  S  V  E  G  R  F  T  I  S  R  D  N  A  K  N  S
GACTACGCCGACAGCGTCGAGGGACGTTTTACAATCTCGCGCGATAATGCGAAAAACTCC
L  Y  L  Q  M  N  S  L  R  A  E  D  T  A  V  Y  Y  C  A  K
CTTTATTTACAAATGAATAGTTTGCGCGCCGAGGACACTGCGGTTTATTATTGTGCCAAA
V  S  Y  L  S  T  A  S  S  L  D  Y  W  G  Q  G  T  L  V  T
GTGTCATATTTGTCCACCGCTTCTTCTTTAGACTATTGGGGTCAGGGAACGTTAGTTACG
V  S  S  S  G  G  G  G  S  G  G  G  G  S  G  G  G  G  S  D
GTAAGCAGTTCAGGAGGTGGGGGGTCTGGGGGCGGCGGCAGTGGAGGTGGTGGATCCGAT
I  Q  M  T  Q  S  P  S  S  L  S  A  S  V  G  D  R  V  T  I
ATCCAAATGACTCAGTCGCCGTCAAGCCTTAGCGCGTCTGTGGGAGATCGTGTCACAATC
T  C  R  A  S  Q  G  I  R  N  Y  L  A  W  Y  Q  Q  K  P  G
ACCTGTCGTGCTTCGCAAGGTATCCGTAATTATTTGGCCTGGTATCAGCAGAAACCGGGA
K  A  P  K  L  L  I  Y  A  A  S  T  L  Q  S  G  V  P  S  R
AAAGCACCCAAGCTTCTGATTTACGCGGCATCTACACTTCAGTCCGGCGTACCCTCACGT
F  S  G  S  G  S  G  T  D  F  T  L  T  I  S  S  L  Q  P  E
TTCTCGGGGTCAGGATCAGGAACTGACTTTACATTGACTATTAGTTCTCTGCAGCCGGAA
D  V  A  T  Y  Y  C  Q  R  Y  N  R  A  P  Y  T  F  G  Q  G
GACGTTGCTACATATTATTGTCAGCGCTACAACCGCGCACCTTACACATTCGGACAGGGT
T  K  V  E  I  K  G  T  G  G  S  G  G  S  L  P  E  T  G  G
ACGAAGGTTGAGATCAAGGGTACCGGGGGCTCTGGGGGGTCGCTGCCTGAGACAGGTGGT
H  H  H  H  H  H  *
CACCACCATCATCATCATTAA
```

PelB sequence

Heavy chain

Light chain

His tag

Figure S17. DNA and corresponding amino acid sequence of V_HV_L format adalimumab scFv.

```
M  K  Y  L  L  P  T  A  A  A  G  L  L  L  L  A  A  Q  P  A
ATGAAGTATTTATTGCCAACCGCCGCCGCCGGCCTTCTGTTGTTGGCCGCACAACCCGCA
M  A  D  I  Q  M  T  Q  S  P  S  S  L  S  A  S  V  G  D  R
ATGGCAGATATCCAAATGACTCAGTCGCCGTCAAGCCTTAGCGCGTCTGTGGGAGATCGT
V  T  I  T  C  R  A  S  Q  G  I  R  N  Y  L  A  W  Y  Q  Q
GTCACAATCACCTGTCGTGCTTCGCAAGGTATCCGTAATTATTTGGCCTGGTATCAGCAG
K  P  G  K  A  P  K  L  L  I  Y  A  A  S  T  L  Q  S  G  V
AAACCGGGAAAAGCACCCAAGCTTCTGATTTACGCGGCATCTACACTTCAGTCCGGCGTA
P  S  R  F  S  G  S  G  S  G  T  D  F  T  L  T  I  S  S  L
CCCTCACGTTTCTCGGGGTCAGGATCAGGAACTGACTTTACATTGACTATTAGTTCTCTG
Q  P  E  D  V  A  T  Y  Y  C  Q  R  Y  N  R  A  P  Y  T  F
CAGCCGGAAGACGTTGCTACATATTATTGTCAGCGCTACAACCGCGCACCTTACACATTC
G  Q  G  T  K  V  E  I  K  S  S  G  G  G  G  S  G  G  G  G
GGACAGGGTACGAAGGTTGAGATCAAGAGTTCAGGAGGTGGGGGGTCTGGGGGCGGCGGC
S  G  G  G  G  S  E  V  Q  L  V  E  S  G  G  G  L  V  Q  P
AGTGGAGGTGGTGGATCCGAGGTGCAGCTGGTTGAATCTGGCGGTGGTCTTGTTCAACCC
G  R  S  L  R  L  S  C  A  A  S  G  F  T  F  D  D  Y  A  M
GGACGTTCTTTGCGTTTGTCTTGCGCTGCGTCGGGTTTCACCTTCGACGACTATGCCATG
H  W  V  R  Q  A  P  G  K  G  L  E  W  V  S  A  I  T  W  N
CATTGGGTTCGCCAGGCTCCAGGTAAAGGGCTGGAGTGGGTATCCGCGATTACCTGGAAC
S  G  H  I  D  Y  A  D  S  V  E  G  R  F  T  I  S  R  D  N
TCTGGTCATATTGACTACGCCGACAGCGTCGAGGGACGTTTTACAATCTCGCGCGATAAT
A  K  N  S  L  Y  L  Q  M  N  S  L  R  A  E  D  T  A  V  Y
GCGAAAAACTCCCTTTATTTACAAATGAATAGTTTGCGCGCCGAGGACACTGCGGTTTAT
Y  C  A  K  V  S  Y  L  S  T  A  S  S  L  D  Y  W  G  Q  G
TATTGTGCCAAAGTGTCATATTTGTCCACCGCTTCTTCTTTAGACTATTGGGGTCAGGGA
T  L  V  T  V  S  G  T  G  G  S  G  G  S  L  P  E  T  G  G
ACGTTAGTTACGGTAAGCGGTACCGGGGGCTCTGGGGGGTCGCTGCCTGAGACAGGTGGT
H  H  H  H  H  H  *
CACCACCATCATCATCATTAA
```

PelB sequence

Heavy chain

Light chain

His tag

Figure S18. DNA and corresponding amino acid sequence of V_LV_H format adalimumab scFv.

Table S1. Affinity of protein M WT and M-8his for Adalimumab scFv (containing a kappa light chain) and CR6261 scFv (containing a lambda light chain) in both the V_H-V_L and V_L-V_H format. K_D values are calculated from titration experiments in Fig. S4 (Adalimumab scFv) and Fig. S5 (CR6261 scFv) which were performed at room temperature and pH 7.4.

	scFv formate	Protein M WT K_D (nM)	M-8his K_D (nM)
**Adalimumab scFv (kappa)**	V_H-V_L	0.37 ± 0.02	2.1 ± 0.08
	V_L-V_H	4.4 ± 0.2	49 ± 8
**CR6261 scFv (lambda)**	V_H-V_L	0.13 ± 0.01	15.0 ± 0.4
	V_L-V_H	0.24 ± 0.01	51 ± 3

Table S2. Association and dissociation rate constants (s^{-1}) of indicated protein M mutants for immobilized cetuximab Fab determined by SPR at pH 7.5 (from data shown in Figure S6).

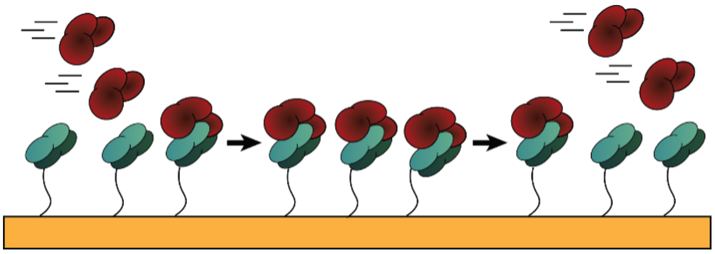

Protein M association and dissociation rates

	k_a (M^{-1} s^{-1})	k_d (s^{-1})	K_D (nM)
**pM WT**	7.93 ± 0.009 $\cdot 10^4$	5.91 ± 0.03 $\cdot 10^{-5}$	0.75
**390H/394H**	6.01 ± 0.010$\cdot 10^4$	<1$\cdot 10^{-5}$	<0.16
**144H/158H/341H/390H/429H**	4.13 ± 0.003$\cdot 10^4$	<1$\cdot 10^{-5}$	<0.23
**144H/158H/394H/429H**	2.26 ± 0.002$\cdot 10^4$	<1$\cdot 10^{-5}$	<0.44
**pM8xHis**	3.46 ± 0.003$\cdot 10^4$	3.33 ± 0.02$\cdot 10^{-5}$	0.96

Table S3. Dissociation rate constants (s^{-1}) of indicated protein M mutants for immobilized cetuximab Fab determined by SPR at indicated pH.

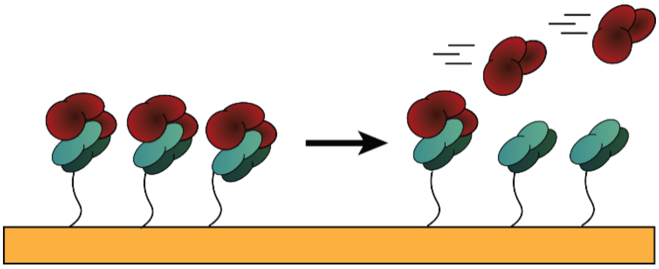

	pH 7.5	pH 6.0	pH 5.0	pH 4.0	pH 3.0
**pM WT**	5.91 ± 0.03 $\cdot 10^{-5}$	n.d.	9.17 ± 0.66$\cdot 10^{-5}$	1.70 ± 0.08$\cdot 10^{-4}$	1.92 ± 0.08$\cdot 10^{-4}$
**390H/394H**	9.13 ± 0.38$\cdot 10^{-6}$	n.d.	1.27 ± 0.08$\cdot 10^{-4}$	4.04 ± 0.92 $\cdot 10^{-3}$	5.73 ± 0.17 $\cdot 10^{-2}$
**144H/158H/341H/ 390H/429H**	4.42 ± 0.15$\cdot 10^{-6}$	n.d.	1.64 ± 0.29$\cdot 10^{-4}$	6.38 ± 0.14$\cdot 10^{-2}$	>0.1
**144H/158H/394H/ 429H**	5.51 ± 0.14 $\cdot 10^{-6}$	n.d.	3.25 ± 0.13 $\cdot 10^{-3}$	>0.1	n.d.
**pM8xHis**	3.33 ± 0.02$\cdot 10^{-5}$	8.28 ± 0.28$\cdot 10^{-4}$	6.33 ± 0.25$\cdot 10^{-2}$	n.d.	n.d.

References

[1] Y. Ni, B. J. H. M. Rosier, E. A. van Aalen, E. T. L. Hanckmann, L. Biewenga, A.-M. M. Pistikou, B. Timmermans, C. Vu, S. Roos, R. Arts, W. Li, T. F. A. de Greef, M. M. G. J. van Borren, F. J. M. van Kuppeveld, B.-J. Bosch, M. Merkx, A plug-and-play platform of ratiometric bioluminescent sensors for homogeneous immunoassays, *Nat Commun* **2021**, *12*, 4586.

[2] G. A. O. Cremers, B. J. H. M. Rosier, A. Meijs, N. B. Tito, S. M. J. van Duijnhoven, H. van Eenennaam, L. Albertazzi, T. F. A. de Greef, Determinants of Ligand-Functionalized DNA Nanostructure–Cell Interactions *J Am Chem Soc* **2021**, *143*, 10131–10142.

[3] Boshuizen, J.; Koopman, L. A.; Krijgsman, O.; Shahrabi, A.; van den Heuvel, E. G.; Ligtenberg, M. A.; Vredevoogd, D. W.; Kemper, K.; Kuilman, T.; Song, J.-Y.; Pencheva, N.; Mortensen, J. T.; Foppen, M. G.; Rozeman, E. A.; Blank, C. U.; Janmaat, M. L.; Satijn, D.; Breij, E. C. W.; Peeper, D. S.; Parren, P. W. H. I. Cooperative Targeting of Melanoma Heterogeneity with an AXL Antibody-Drug Conjugate and BRAF/MEK Inhibitors. *Nat. Med.* **2018**, *24*, 203– 212.

[4] R. K. Grover, X. Zhu, T. Nieusma, T. Jones, I. Boero, A. S. MacLeod, A. Mark, S. Niessen, H. J. Kim, L. Kong, N. Assad-Garcia, K. Kwon, M. Chesi, V. v Smider, D. R. Salomon, D. F. Jelinek, R. A. Kyle, R. B. Pyles, J. I. Glass, A. B. Ward, I. A. Wilson, R. A. Lerner, A Structurally Distinct Human Mycoplasma Protein that Generically Blocks Antigen-Antibody Union *Science* **2014**, *343*, 656–661.
